# How ‘who someone is’ and ‘what they did’ influences gossiping about them

**DOI:** 10.1371/journal.pone.0269812

**Published:** 2022-07-06

**Authors:** Jeungmin Lee, Jerald D. Kralik, Jaehyung Kwon, Jaeseung Jeong

**Affiliations:** 1 Department of Bio and Brain Engineering, College of Engineering, Korea Advanced Institute of Science and Technology (KAIST), Daejeon, Republic of Korea; 2 Program of Brain and Cognitive Engineering, College of Engineering, Korea Advanced Institute of Science and Technology (KAIST), Daejeon, Republic of Korea; University of Edinburgh, UNITED KINGDOM

## Abstract

To understand, predict, and help correct each other’s actions we need to maintain accurate, up-to-date knowledge of people, and communication is a critical means by which we gather and disseminate this information. Yet the conditions under which we communication social information remain unclear. Testing hypotheses generated from our theoretical framework, we examined when and why social information is disseminated about an absent third party: i.e., gossiped. Gossip scenarios presented to participants (e.g., “Person-X cheated on their exam”) were based on three key factors: (1) target (ingroup, outgroup, or celebrity), (2) valence (positive or negative), and (3) content. We then asked them (a) whether they would spread the information, and (b) to rate it according to subjective valence, ordinariness, interest level, and emotion. For ratings, the scenarios participants chose to gossip were considered to have higher valence (whether positive or negative), to be rarer, more interesting, and more emotionally evocative; thus showing that the paradigm was meaningful to subjects. Indeed, for target, valence, and content, a repeated-measures ANOVA found significant effects for each factor independently, as well as their interactions. The results supported our hypotheses: e.g., for target, more gossiping about celebrities and ingroup members (over strangers); for valence, more about negative events overall, and yet for ingroup members, more positive gossiping; for content, more about moral topics, with yet all domains of social content communicated depending on the situation—context matters, influencing needs. The findings suggest that social knowledge sharing (i.e., gossip) involves sophisticated calculations that require our highest sociocognitive abilities, and provide specific hypotheses for future examination of neural mechanisms.

## Introduction

A 400-year-old line by John Donne says, “No man is an island entire of itself”—people need each other. Without others, the extent of our knowledge and achievements is limited to our own experiences and abilities; expanding exponentially by sharing our experiences and collaborating with others (in current time and across history). And knowledge sharing importantly includes information about each other. That is, to negotiate our multi-agent world, we must properly understand and predict others’ actions, which in turn requires accurate, up-to-date knowledge of them [1–5]; and because so much social activity takes place in our absence, communicating this social information is necessary. Indeed, even beyond prediction, social communication can bootstrap society’s means to monitor, teach, regulate, and limit deleterious effects of improper behavior and social interaction.

In fact, roughly two thirds of daily conversation appears to involve social information sharing [6, 7], with the majority of such free conversations about people not present at the scene [8]. When we communicate to others (i.e., sender to receiver) about an absent target, we call it *gossip* [see 9, 10]. Gossip is generally thought to be trivial, so-called "tabloid fodder," mere entertainment, or else of malicious intent (such as ‘badmouthing’ rivals) [4, 9, 11–20]. Further, it is often considered an undesirable behavior that violates solidarity norms by “talking behind one’s back” instead of direct confrontation [20, 21]. Yet the need for social information sharing suggests that it should occupy an important position in human interactions. To what extent, then, is gossiping—talking about absent others—based on critical functional needs versus other more superficial or malicious reasons?

### Previous findings

Studies indeed suggest that gossip plays an important role in our society. The three main functions supported by literature are (1) information dissemination, (2) group protection and social control, and (3) social bonding, which we consider in turn [e.g., 2, 3, 9, 14, 22–27]. For information dissemination, De Backer et al. [28], for example, suggested two general types of information-driven gossip: strategy learning and reputation [also see 29]. For the first, strategy learning, people engage in gossiping (sharing and consuming) to learn fitness-relevant information such as social norms (that show how to behave appropriately within a group) [see also 3], mating strategies (i.e., information about mating and parenting), and survival or life know-how (especially related to universals, such as not swimming with sharks). This type of information is based on the content of the event gossiped about (i.e., what the target was involved in), and largely independent of the target her/himself. Information-driven reputation gossip, in contrast, centers on the target person of the gossip, and involves using the episode to learn basic characteristics about them (such as whether trustworthy or not). The latter, then, would be presumed more valuable if the target is someone close to the sender and/or receiver compared to, for example, a stranger. They further suggest that for celebrities, heightened interest in them suggests a “parasocial” sense of closeness to or intimacy with them, and found evidence for this (especially in older people; while finding younger people more interested in learning life lessons from the content—i.e., strategy learning) [28, 30, 31]. Multiple other studies have also found evidence for the importance of information gathering, and therefore learning, via gossip [2, 3, 32–34].

The second main function of gossip relates to the reputation information, but emphasizes using or updating it (based on a current event) to actively maintain social norms, promote cooperation, and when necessary, respond to improper behavior (e.g., free-riders) by warning others and/or sanctioning the behavior [25, 35–42; see 26, 27, 43–45 for recent reviews and evidence for the gossip-cooperation relationship]. Sanctioning can run the gamut from avoiding or ostracizing to finding a receiver that can directly confront the target person. Thus, gossip can serve as an effective tool for group protection and social control [9, 25, 35–42, 46–48].

For the third main function, even in cases that may seem superficial, gossip appears to serve as an important tool for social bonding to connect the two people who share it (i.e., gossiper and receiver). In his popular book *Grooming*, *Gossip and the Evolutionary Language*, Dunbar [23] considers human gossip behavior as analogous to and even possibly stemming from primate grooming behavior, arguing that gossip produces interpersonal trust that strengthens dyadic bonds. In support, gossip does appear to promote friendship, interpersonal trust and closeness [2, 34, 49–53].

Besides the main three functions of gossip, researchers have found evidence for others as well. For example, reviewing the gossip literature to date, Foster [9] categorized the evidence into four main functions of gossip: the three described here, along with entertainment. That is, besides the other functions, gossip appears as well to simply entertain, such as via arousing our general interests in problem-solving, drama, vicarious experiences, and (favorable) social comparisons [4, 9, 16–19].

In a similar vein, Beersma and Van Kleef [24] examined four distinguishable motives: information gathering and validation, group protection (focusing on warning others), negative influence, and social enjoyment; the latter of which potentially includes both social bonding and entertainment. Moreover, in their case, ‘negative influence’ focused on purposely attempting to denigrate others as “indirect aggression.” Indeed, this may capture the intuition that gossip can be used to purposely denigrate others, such as one’s rivals. In general, they found evidence for all four motives, with yet the informational one being more prevalent than the others; though in a condition where the target violated the norm (i.e., did not cooperate), group protection proved especially strong.

Building on the four motives examined by Beersma and Van Kleef [24], Dores Cruz et al [54] developed a questionnaire that directly asked participants to rate the extent to which each motive drove their last remembered gossiped item. Together with these four, they found evidence for a fifth also tested: emotional venting (such as gossiping to feel better, let off steam, etc.) [55, 56].

Table 1 provides a summary of these six functions of gossip that have received significant empirical support. In short, there are clearly important functional reasons to gossip; and at the same time, some evidence also supports popular notions of being superficial (such as for mere entertainment), malicious, and emotionally driven.

**Table 1:** Summary of main motives to gossip (from literature).

1. Information dissemination: gathering, learning, sharing, validating • World knowledge • Survival & Achievement (i.e., for basic living, economic/career, skills, expertise) • Mating (i.e., information about mating and parenting) • Social (e.g., norms, ethics, power, prestige, influence) • Entertainment related (e.g., activities to try) • Person knowledge • More stable characteristics (e.g., personality, reputation/status) • Day-to-day (i.e., up-to-date news about them)
2. Group protection, Social control, Reputation • To warn others • To change target’s behavior: via reputation or more direct receiver confrontation
3. Social bonding (e.g., sharing beliefs, secrets) • For alliances, friendships (real and parasocial) • For self-promotion (to receiver and/or target)
4. Enjoyment/Entertainment (e.g., drama, vicarious experience, self-comparison)
5. Negative influence (with manipulative intent to denigrate the target)
6. Emotion venting (e.g., to feel better)

Along with main motives to gossip, other *contextual* factors also influence gossiping, such as the relationships among the gossip target, gossiper, and receiver. In reviewing the gossip literature, Wittek and colleagues, for example, single out the specific interdependences between the gossip triads (i.e., gossiper, receiver, and target) as critical social context that must influence gossip behavior [57, 58]. For instance, Wittek & Wielers [57] found that gossip in both schools and workplace appears to be especially promoted when the gossiper and receiver share a close relationship, and together have poor relationships with the target; and this was opposed to cases when all three have good relationships with each other. Yet others have found evidence of positive gossip spreading that suggest evidence for the latter [8, 32, 57, 59–61]. Clearly, then, more testing is necessary to flesh out the influence of the relationships on gossiping.

Another critical relational factor is broadly construed as *social-structural* by Giardini & Wittek [26], and includes such things as the relative status among the parties. One way to examine this is with celebrities as targets of gossip, having fame and influence. For example, one study found that adolescents with less experience consider celebrities prestigious with high social status and try to learn life strategies for success [28]; while another found that adolescent women tend to be harsh on celebrities and consider bashing and negative critiquing as the price to pay for their fame and status [62]. However, more work is needed to fully understand the relationship between status and gossip, which we do as well in the current study.

Finally, for contextual factors, other critical ones include (a) the individual characteristics of the three parties (like personality traits) and (b) *cultural-institutional* ones, such as setting (of target and gossiping events) (e.g., workplace, school, home, public place) or group memberships among the parties (e.g., shared community, organization, culture, and society) [26, 45, 63–69]. The effects of many of these have yet to be thoroughly examined, though outside the scope of the current study [26].

Along with motives to gossip, there are obviously potential costs. Giardini and Wittek [20, 26], in particular, built an analytical framework focusing directly on why people *do not* gossip, providing reasons to refrain based on ‘functional interdependence’ (i.e., senders strategically withhold the information for gains) and ‘socio-affective interdependence’ (i.e., refrain because they do not want to harm the relationship among gossip dyads: sender-receiver, sender-target, and receiver-target).

Moreover, examining Table 1 it is clear that there are potentially multiple competing motives for any given event one might gossip. To account for this, in their analytical framework Giardini and Wittek [20, 26] adopted goal framing theory [70]. According to the theory, there are three goal frames in which only one goal becomes salient in a given situation: the *hedonic goal frame* that wants to “feel good” at the moment of decision; the *gain goal frame* that wants to protect preexisting or to earn new resources; and finally, the normative goal frame that wants to conform to particular social norms. The three frames are arranged in a hierarchy with the hedonic goal being the strongest, the gain goal second, and the normative goal last. Their main argument is that gossip is ubiquitous in our daily life chiefly because it gives us hedonic gratification–i.e., it is inherently enjoyable [4, 9, 14–19, 23, 71]. Nonetheless, we may also gossip due to gain and norm goals outcompeting the hedonic one. Moreover, when either of the latter two ‘win’, they may also lead to refraining from gossiping. As noted above, the authors also emphasize the relationships among the gossip triads (i.e., gossiper, receiver, and target); and as such, depending on the arrangement of the goal frames (i.e., which goal becomes salient in a given condition) and the relationship between the three actors in the gossip triad, the benefits and cost of spreading gossip are decided. If the cost is too high, the gossip is inhibited.

Although this recent theoretical framework is based on reviewing the literature without directly testing it yet, there are many important elements, including recognizing competing goals, with each having corresponding potential benefits and costs, which in turn are also dependent on contextual factors such as the relationships among the gossip target, gossiper, and receiver. In fact, we believe that the current state of findings can benefit from an even more comprehensive model along the same lines, and one based more directly on the human mind/brain: i.e., the comprehensive set of processes underlying gossiping behavior. To this end, we have been developing such a model [72–75]. It shares general characteristics with goal framing theory, including goals, frames or problem representations built around them, and competition among them. A mind/brain-based model also helps to clarify more enigmatic but significant factors such as (a) the role(s) played by emotion (and how it relates to underlying functional motives such as survival, mating, etc.); (b) how benefits and costs related to each goal influence the decision to gossip or not; (c) accommodating even larger sets of goals that compete to control gossip behavior (e.g., from Table 1); (d) larger range of possible events (i.e., event content) to potentially gossip; and so on.

For example, a key next step in gossip research is to examine how multiple social events may trigger different gossip motives and lead to potential gossip behavior. Although many empirical studies have investigated the relationship between social norms and gossip [25, 35–42], most have focused on cooperation. Although cooperation (or fairness) is indeed one of the most fundamental elements of society, and broadly construed covers a wide range, gossip enables people to learn about all culturally accumulated social norms and teaches them how to behave appropriately in given social contexts [3, 28]. As such, social norms cover many more categories of socially accepted rules of behavior, many of which are also fundamental and moral: e.g., those from moral foundations theory, ranging from prosociality to purity [76–79]. With respect to morality, researchers have indeed established its importance in driving and interpreting gossip behavior, but only via a small subset of cases [64–66, 68].

At the same time, a high portion of daily gossip contains other social content such as personal habits, looks, interests, and achievements [6, 8]. Thus, to more fully cover the content of daily gossip, both norm-related and more general, person-related gossip should be examined together. This in turn requires a comprehensive framework that enables *a priori* predictions of when and why wide-ranging social information would be disseminated: i.e., whether gossiping would occur.

Therefore, we first briefly describe our general framework of social information communication (i.e., gossip), developed to provide a comprehensive description of the internal process of a social agent, which in turn provides specific predictions about how social information is disseminated (i.e., whether gossiped or not). These predictions are then tested in the current study.

### Theoretical framework

The extensive work and findings to date suggest the need to develop a framework that models the human mind/brain, and one that provides enough sociocognitive detail necessary to make accurate *a priori* predictions about specific human social interactions, and in particular, whether someone will gossip a given piece of social information, across a wide array of circumstances. We present a scaled-down version most relevant to the current study [72–75].

**Fig 1:**
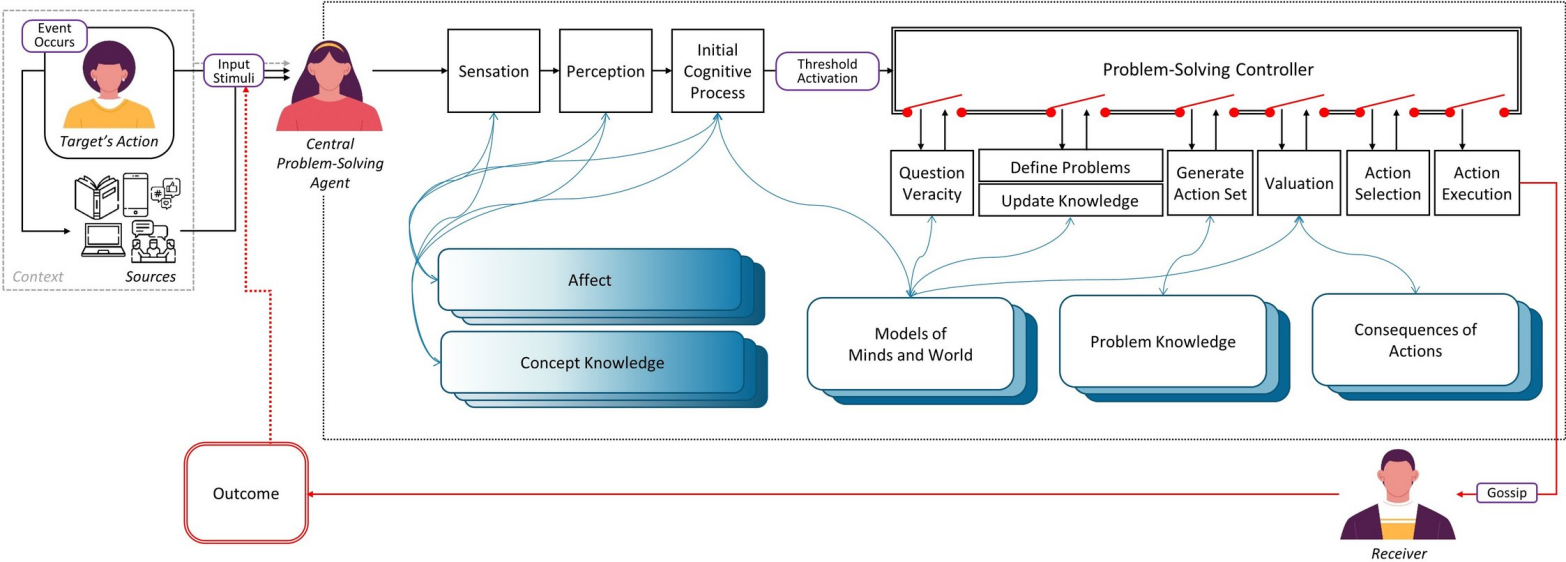
Our framework of social information processing. The central agent goes through a series of internal processes (black rectangles) after learning about some event involving a target individual, which includes accessing and updating relevant knowledge (blue rounded rectangles), and determining whether to take action about it. See text and Lee, Kralik, Jeong [72–75] for details. (Three face images from: Freepik.com).

Depicted in Fig 1, our general framework centers on a focal problem-solving agent, the central agent, as a member of a multi-agent system, interacting with others, building a sophisticated network of relationships, and making decisions about the best actions to take to ultimately maximize outcome. (To avoid “his/her” we denote the central agent as a woman.) We focus on instances in which someone else—a target person—is involved in some incident that the central agent learns about (via direct observation or indirect communication). We then model how the central agent processes this information, both in terms of updating her own knowledge about the target person and event, and then whether she should do anything about it: specifically, whether to gossip. To clarify how the theoretical framework was used to generate hypotheses for the current study, we next (a) describe the main factors processed by the model and examined in the current study (content, valence, and target); and then (b) describe how the model processes this information to determine whether or not to gossip (see [72] for more detailed description).

#### Content and its valence

**Fig 2:**
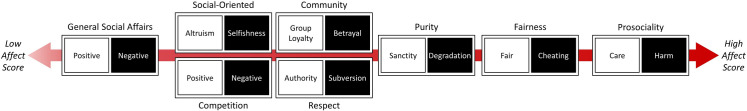
Social domains used in the current study. Eight different social domains (with their positive and negative components) used in the current study and their relative positions from lower to higher significance, in terms of right and wrong, good and bad behavior, and potential impact. Five morality domains (prosociality, fairness, community, respect, and purity) were adopted and modified from the moral foundations theory [76–79]. The remaining three (competition, social-oriented, and general social affairs) were selected to represent other social activities.

In this and the following section we present the main types of social information examined in the current study. Fig 2 shows the eight different content domains we used to produce scenarios that attempt to span the space of possible social events. Five were adopted from the moral foundations theory (prosociality, fairness, community, respect, and purity modified from the five universal moral foundations suggested by the theory: harm/care, fairness/reciprocity, ingroup/loyalty, authority/respect, and purity/sanctity) [see 76–79]; another (general social affairs) to represent other more everyday social activities (e.g., seeing a movie); and an additional two (competition, social-oriented) to cover events somewhat in between general social affairs, with, for example, a weaker sense of right vs. wrong [6, 8].

More specifically, based on prior evidence and theoretical considerations, in the model we place the eight social domains on two main scales [see 6, 8, 72–79]. The first, called the *morality-content scale*, is represented in Fig 2, and is based on more universal issues of right and wrong, good and bad behavior—thus, essentially a morality or social norm scale related to the most evolutionarily relevant social information, such as for group protection and social control. The second content scale is a person-knowledge dimension, reflecting the degree the event activity provides useful information about the target, such as their preferred hobbies, activities, and relationship status (similar to De Backer et al.’s, [28], reputation information). This information, whether reflecting more stable characteristics or more dynamic “day-to-day” ones (e.g., weekend activities of the target, such as fishing, baking cookies), has the potential to be significant depending on who the target is because they nonetheless are important for successful day-to-day social interaction. On this second scale, the scores in Fig 2 are essentially flipped, with general social affairs the highest, followed by social-oriented and competition, etc.

Additionally, events usually carry a *valence*, whether positive (e.g., helping others) or negative (e.g., causing harm). And as previous studies suggest, gossip is never always negative or positive—with some even finding both at comparable rates [8, 57, 59]. Hence, in our framework, each content domain (for both scales) consists of positive and negative counterparts, and thus its corresponding valence. For example, prosociality is divided into negative harm (e.g., “Sam stabbing a person with a knife”) and its positive counterpart care (e.g., “Alex saving a child from a fire”) (see Method for details). Hence, the second main factor examined is the content valence.

#### Target

The third main factor examined in the study is the identity of the target, i.e., who was involved in the incident. We recognize three key components underlying target identity that would be expected to influence potential gossiping by the central agent. The first is the degree of *intimacy*: i.e., how physically and emotionally close the central agent feels to the target. The second is the level of *interest* the central agent has for the target individual: i.e., how much do the central agent and their social network want to know about the detailed exploits of the target individual. And the third, the degree of the target’s social *influence*: i.e., how many agents within the society are potentially impacted by the target and their actions.

To examine these three factors (influence of the target person, and interest and intimacy of the central agent toward them), as shown in Table 2, we used three target groups that differentiated among them: (1) *ingroup* (i.e., close friends who are in the same social group with the central agent), (2) *outgroup* (i.e., a stranger whom the central agent has never met), and (3) *celebrity* (i.e., people with high influence, fame, reputation and/or popularity like actors, pop stars, sports stars, etc.). We note that outgroup can be defined in various ways, such as being anyone with whom the central agent does not identify, which could include others who are not necessarily strangers. However, for tighter control of the underlying factors we focus on only those who have never interacted with the central agent and are never expected to. Thus, such outgroup people would have almost no influence on the central agent in terms of possible future social interaction, and therefore are considered strangers.

**Table 2:** Three main components differentiate the target groups. Ingroup members (i.e., close friends) are expected to be high in intimacy and interest, but relatively low in influence; outgroup members (i.e., strangers) low in all categories; and celebrities high in influence, midlevel in interest, and low in intimacy.

	*Intimacy*	*Interest*	*Influence*
Ingroup	High	High	Low
Outgroup	Low	Low	Low
Celebrity	Low	Mid	High

#### From input to initial cognitive processing

We are now in position to describe how the three main factors–content, valence, and target–are processed by the model. As shown in Fig 1, information about a social event is received from an external source (e.g., via friends, mass or social media, etc.) and first passes through *Sensation* (e.g., auditory or visual) and *Perception* processes. The potential significance–or impact–of the event is then first estimated by the *Initial Cognitive Process* to determine whether additional processing is necessary. This initial assessment is considered an affective-emotional reaction based roughly on what happened (content and its valence) and the person involved (target). For content, the impact of the event (e.g., ‘Tim punched his friend in the face.’; see Methods) is first estimated with two main types of knowledge: *concept knowledge* to understand what “punched” for example means and *emotional knowledge* attached to it (*Affect and Concept Knowledge* in Fig 1). At this stage, the *morality-content scale* (Fig 2) is the only one used to trigger the initial affective response, as it is based on the most evolutionarily relevant social information; and content with a strong sense of morality (e.g., harming someone or cheating others), for example, has been found to be associated with strong emotional responses [80, 81]. In the model, this scale thus determines the *affect score*.

For target, the *Initial Cognitive Process* resolves the target into intimacy, interest, and influence values (as described above). The affect and target (i.e., intimacy, interest, influence) values are then combination to generate *one overall impact value* that must exceed threshold to produce additional event processing. For example, even with relatively low moral impact, such as general social affairs (e.g., "gained 10 pounds last winter"), there can be relatively high total significance when the affect score combines with a high-impact target (e.g., "your best friend Sarah"). Likewise, even for low-impact targets like strangers, the total impact depends as well on the severity of the act, such as regarding harm, the negative component of prosociality (e.g., "stabbed a person with a knife") [4, 28, 29].

#### From threshold activation to decision valuation

If the derived impact value of the information about the event exceeds threshold, the metacognitive *Problem-Solving Controller* module is initiated (double-lined box in Fig 1). The goal of this module is to orchestrate the processes necessary to make a decision on whether or not to spread the received information (based on the potential impact of the event and the expected consequences of a chosen action). After potentially confirming veracity and then updating knowledge, the *Define Problem* subprocess builds a set of ‘problems’ (i.e., goals and their achievement status) that the event has provoked. For the current study, as shown in Table 3, our model recognizes two main goals (G1 and G2) to solve the problems caused by the event. The first goal (G1) is in maintaining social norms, especially in terms of how we interact with and affect one another, and thus in regulating, ultimately controlling the society [3]; and this largely occurs with the target’s behavior warranting an update in their social standing, i.e., their reputation. There are multiple possible reasons (i.e., benefits) to do this, including hoping to modify the target’s behavior (i.e., as indirect punishment or reward), and preparing others for future interactions (such as warning about potential harm or cheating) [24, 25, 35–42, 48]. At the same time, there are potential costs with pursuing this goal: for example, if the information is wrong and/or misleading to receivers, the target’s reputation could be unfairly updated [44, 82]; moreover, this cost could escalate further if the target seeks retaliation [20, 26, 57].

**Table 3:** Two main social goals as problems to solve. Key benefits and costs for achieving the goals are listed.

*Goals*	*Benefits and Costs*
G1: Group protection & Social control	B1: Prepare others for future interactions; Update target’s reputation; Influence target’s behavior
	C1: Wrong/misleading info that may unfairly alter target’s reputation; Target’s potential retaliation
G2: Share useful person knowledge	B2: Update others’ detailed knowledge about the target
	C2: Wrong/misleading info spread to others and causing inaccurate mental models (of individuals, state of world)

The second Goal (G2) derives from people (especially in the local community) not being up-to-date with useful information about the target, such as for example, a recent fight between two friends, which ultimately could cause awkward, inappropriate, or at minimum, inefficient community interactions [6, 8]. The benefit of pursuing this goal is that the people who are informed are all ‘on the same page’ about the target–and thus have accurate knowledge about them and the current state of the world. The main cost of communicating this information, however, would be the potential spreading of inaccurate or misleading information as well, causing the receivers to have inaccurate and thus ‘broken’ models about the target and world state [44, 82].

We also note that the model includes a constant term, that reflects benefits and costs inherent in any social information sharing, such as its social bonding and entertainment values [2, 4, 14–19, 23, 34, 49–53], as benefit, and risk of gaining a bad reputation as a gossiper, or violating the targets’ privacy by spreading personal information, as costs [20, 21]. Thus, the entire set of goals and their associated possible benefits and costs in our model attempts to capture the main social functions and consequences of gossip well-established in past studies (Table 1)–with the current description focused on those most relevant to the current study [9, 10, 20, 23, 24, 26, 28, 57].

#### Valuation and action selection: To gossip or not to gossip

Once the problems and goals are defined, a set of actions to achieve the goals is generated (Fig 1). Our current study focuses on gossip (i.e., the central agent talking to others about the event without the target’s presence)–thus we consider two possible actions: to gossip or not to gossip. The Problem-Solving Controller then moves to *Valuation* to calculate and compare expected-outcome values of each action based on the potential benefits and costs listed in Table 3 across both problems [see 72–75]. Because we consider whether to gossip or not, valuation is simplified to comparing the benefits of gossiping to the costs. That is, the costs of gossiping are equivalent to the benefits of not gossiping–therefore, in this case, the benefits are simply compared to the costs [75]. Thus, to obtain the overall benefit versus overall cost, each benefit (B1 and B2) and cost (C1 and C2) are combined with the relevant target (i.e., intimacy, interest, and influence scores), content (i.e., morality and person-knowledge scores), and valence values, and then all benefits and all costs are summed together respectively. With valuation complete, the Problem-Solving Controller initiates the remaining subprocesses that lead the central agent to select and take the action—in this case, whether to spread the social information or not (to gossip) by comparing the values of overall benefit to overall cost (via a probabilistic softmax process).

### Hypotheses from model predictions

The main hypotheses obtained from the theoretical framework can now be described. Considering each main event factor—the target person, content, and valence—as well as their potential two-way interactions, the model generated six predictions as hypotheses to test in the current study. “B’s” and “C’s” listed below refer to the benefits and costs in Table 3.

#### Target

For the target factor, ingroup information would have high benefit to spread for both reputation (B1) and day-to-day knowledge (B2) updating, due to the high interest in and intimacy with them. Celebrity information as well would be expected to have high benefit, especially given their degree of influence (B1). At the same time, however, ingroup information spreading would be riskier than for celebrities because they are so close to the central agent (C1 & C2), lowering the amount of gossiping expected for ingroup members relative to celebrities. Whereas for outgroup members (i.e., strangers), potential benefits of passing information about them are expected to be low, leading to relatively low gossiping rates. Thus,

Hypothesis1:ForTarget,

Celebrity>Ingroup>>Outgroup

Although limited thus far in number, studies support this hypothesis [9, 83]. In a study with college students, for example, participants were more interested in gossip about allies (including friends) than non-allies (consisting strangers) [83]. Moreover, there is also evidence that status influences gossiping behavior [28, 49, 83]. Nonetheless, examination across a wider range of content domains is needed to better clarify the conditions underlying these potential effects, and especially of the impact of status on information sharing.

#### Valence

Because negative information tends to be more related to threat and potential loss, the benefits to sharing information about it are potentially high: especially, for example, warning others and influencing the target’s behavior (B1). And even though (a) positive incidents about others (such as how caring and helpful) are beneficial to know (B1 & B2), and expected to be significantly gossiped as well, and even with (b) some increased potential cost in spreading negative information (C1 & C2), negative incidents are still expected to be spread more than positive ones. Thus,

Hypothesis2:ForValence,

Negative>Positive>>0

For scenario valence, not only does popular sentiment suspect heightened gossiping of negative events, there also is substantial evidence for it; whereas, the evidence highlighting gossiping about positive scenarios is much more limited [16, 49, 83–86]. It is therefore important to test a wide-range of cases to clarify valence effects.

#### Content

Events involving moral issues are potentially more consequential (e.g., related to harm, fairness or cheating), and thus relaying information about them are expected to provide more benefit to others as well as to the central agent (especially high B1, overriding the high but lower C1). Thus, the first prediction with content is that moral dimensions will be spread more than others, and in particular, more than general social affairs. Then more specifically, a stronger hypothesis might directly track the scale depicted in Fig 1, though we considered this too specific for our current dataset; short of this, due to their heightened importance, the two highest moral dimensions are expected to be most spread: prosociality (most obvious when considering harm) and fairness (as when considering cheating or free riding) [79, 87–89]. Therefore, there are two content predictions:

Hypothesis3:ForContent,

(a)Morality>GeneralSocialAffairs

(b)Prosociality>Fairness>[Theothers]>GeneralSocialAffairs

Multiple studies have indeed found evidence for moral underpinnings of gossip [25, 35–42, 64–66, 68]; however, research has thus far focused narrowly on one or a few moral dimensions (e.g., fairness or purity), and thus a more comprehensive and systematic examination is needed. Indeed, our predictions provide hypotheses for more comprehensive empirical studies. Thus, although the moral dimensions are generally expected to generate more gossip than others, our model has enabled a more specific hypothesis about it.

#### Target-by-valence

If the target is high in influence, the model predicts more spreading of negative incidents because their behavior can potentially adversely affect others so much (both directly, being in contact with more people, and more influential people; and indirectly, for example, as norm setters, role models) (increasing B1); moreover, he/she must deserve such influence, and thus any transgressions are particularly glaring and reacted to. At the same time, the costs are generally expected to be low (C1), with less likelihood of repercussions (especially for a central agent with low relative influence). And although positive incidents involving influential people are expected to garner interest, it would not be as much as negative ones (based on the above). Thus, ‘Celebrity-Negative > Celebrity-Positive’ is predicted.

In addition, if the target is high in interest and intimacy (i.e., the central agent and target have a close relationship), the potential benefits (B1 & B2) of spreading information about them is significant (as they are important in the central agent’s life); yet, especially for targets high in intimacy, the potential costs (mostly C1) could be substantial, even potentially ruining the relationship. And these heightened costs would be expected for negative gossip much more than positive. Thus, the model also predicts ‘Ingroup-Positive > Ingroup-Negative’. Thus, for target-by-valence, the following interaction is predicted:

Hypothesis4:ForTarget‐by‐Valence,

(a)Celebrity‐Negative>Celebrity‐Positive

(b)Ingroup‐Positive>Ingroup‐Negative

Some evidence is consistent with Hypotheses 4 [62, 83, 90, 91]. For example, one study found that people spread positive information about allies and negative information about potential enemies, including strangers and those with high status [90]. But would these results hold up across a wider range of realistic social scenarios?

#### Target-by-content

As events involving moral issues (e.g., care or harm or cheating) are more consequential, it is further expected for these issues to be even more consequential with people of influence (and thus even higher B1). Moreover, with lower intimacy, potential costs would be lower, with repercussions less expected. Indeed, from this, the model predicts that moral incidents (especially for prosociality and fairness) will be spread more often with celebrity targets.

In contrast, if the target is high in interest and intimacy–and especially the latter–greater benefit from maintaining updated day-to-day person-knowledge information about them is expected (like movies they have recently seen; activities engaged in) (B2); with correspondingly less costs in spreading this content (given its less-charged nature) (C2). Therefore, for Target-by-Content, Hypothesis 5 is the following:

Hypothesis5:ForTarget‐by‐Content,

(a)Celebrity‐Prosociality>Ingroup‐Prosociality>>Outgroup‐Prosociality

(b)Celebrity‐Fairness>Ingroup‐Fairness>>Outgroup‐Fairness

(c)Ingroup‐General‐Social‐Affairs>Celebrity‐Same>>Outgroup‐Same

In other words, the predictions highlight the fact that context should matter; and attesting to the importance of developing a comprehensive model of social interaction and communication to specify potentially important context effects.

#### Content-by-valence

As stated above for content, incidents with larger potential ramifications, as well as clearer boundaries between right vs. wrong, would be expected to be spread more. These are the moral ones, and especially prosociality and fairness. And when these incidents are of negative valence, such as harm and cheating, one might expect a higher need for warning others and punishing the problematic behaviors (i.e., particularly high B1). The model thus, in general, predicts higher spreading of the negative component of the moral domains (e.g., harm for prosociality) over their positive counterparts (e.g., care for prosociality) (as described in Hypothesis 2). But given the range in intensity or degree of the moral domains (as captured by the morality-content scale, Fig 1), the study’s hypothesis focuses on the two domains with highest score, and thus, predicting ‘Prosociality-&-Fairness-Negative > Prosociality-&-Fairness-Positive’. That is,

Hypothesis6:ForContent‐by‐Valence,

(a)Harm>Care>>0

(b)Cheating>Fairness>>0

We also note that even though we expect higher spreading rates for the negative dimensions, and in particular, of these two moral domains, the modeling also nonetheless shows that the positive dimensions (for any of the content domains) should matter, as they too are expected to yield benefits above their expected costs. Thus, it is also predicted that all spreading rates will be significantly above zero.

The current study was thus designed to understand the key drivers of social information spreading (i.e., gossiping), based on an event’s target individual, its content, and valence; and more specifically, we sought to test the six hypotheses generated from our model of communication about social information, to explain and predict gossiping behavior.

## Method

### Participants

A total of 104 participants (59 women and 45 men) with no reported history of neurological or psychological disorders were recruited from the local community around the Daejeon area via college bulletin boards and online recruitment websites. The performance of every participant was monitored carefully during the experiment; two of the 104 participants were excluded from data analysis for falling asleep during experimental sessions. As a result, data acquired from 102 participants (59 women and 43 men, mean age 23.8 years, range 20–32) were analyzed. The experimental procedures were approved by the Institutional Review Board (IRB) of Korea Advanced Institute of Science and Technology (KAIST) prior to the study. For participant consent, everyone was first provided with an informed consent form (that was also reviewed and approved by the IRB of KAIST). While each participant read the consent form, the experimenter was present to answer any questions. Once going through the form thoroughly, the participant then decided whether to proceed in the experiment or not (they were also informed that there would be no disadvantage in choosing to discontinue). Participant consent was then obtained with their written signature on the form agreeing to participate.

### Experimental paradigm and ecological validity

We sought to develop a paradigm that best balanced ecological validity and scientific rigor. We thus (a) developed realistic scenarios of target events that yet tested our independent variables systematically; (b) inserted actual names of their close friends for ingroup members (queried pretest), as well as using well-known Korean celebrities; (c) presented the scenarios in short text read on a computer screen (to simulate how information is often acquired and consumed by the participants in daily life); (d) obtained their immediate reactions by first querying whether they would or would not gossip (as simple yes or no); (e) then asked them to rate each scenario on a series of factors to assess the realism of the overall paradigm; and finally (f) queried them directly posttest regarding how realistic it was to them.

### Scenarios

To generate social information for participants, we created hypothetical events caused by a target, called scenarios, described in one or two sentences (see Table 4). Each scenario was categorized using three factors: (1) target identity (i.e., identity of the third party who is the subject of the given gossip), (2) valence (i.e., polarity of each gossip in terms of good and bad, or right and wrong), and (3) content (i.e., the type of information that each gossip contains). *Target identities* were divided into three types: ingroup, outgroup, and celebrity. Prior to the task, participants were asked to submit five names of their close friends, with the names inserted in the scenarios for ingroup targets. For outgroup gossip, names used as outgroup members were chosen to be rare enough so that participants would not be familiar with them. To provide additional certainty that the outgroup members were truly so, regions (or hometowns) of the outgroup targets were also included in the scenarios, with regions that were rare and obscure to the Korean participants. For celebrity gossip, we used the names of Korean celebrities including famous actors, actress, singers, sport stars, etc. *Valence* of the scenarios was either positive or negative.

**Table 4:** Gossip categorization and examples. Contents with superscript “m” contain moral aspects based on the moral foundations theory [76–79].

*Factors for categorization*		*Example scenarios*
Target	Ingroup	Your friend, [a name provided by the participant], baked cookies with the neighbor.
	Outgroup	[A random name] living in [a random place] went camping alone last weekend.
	Celebrity	[A famous Korean celebrity] recently broke up with [his/her] [boyfriend/girlfriend].
Valence	Positive	[A target] studied hard for the exam while [his/her] friends were planning to make a cheat sheet.
	Negative	[A target] cheated on the final exam.
Content	Prosociality (Care/Harm)^m^	[A target] jumped down on the subway track to save a person who accidentally fell down. (*Care*)
		[A target] punched a random person on the street in the face. (*Harm*)
	Fairness (Fair/Cheating)^m^	[A target] accidently received a much better grade than [he/she] actually deserved on the final exam due to the TA’s mistake. [A target] went to the professor and willingly had [his/her] mark deducted. (*Fair*) /
		[A target] cheated on the final exam and received a better grade than [he/she] actually deserved. (*Cheating*)
	Purity (Sanctity/Degradation)^m^	[A target] never leaves the washroom before washing [his/her] hands. (*Sanctity*)
		[A target] never washes [his/her] hands. (*Degradation*)
	Community (Group Loyalty/Betrayal)^m^	[A target] is loyal to the club and never says bad things about the members. (*Group Loyalty*)
		[A target] spoke ill of [his/her] mother country in a room full of people from various places around the world during a multicultural party. (*Betrayal*)
	Respect (Authority/Subversion)^m^	[A target] gave [his/her] seat to an elderly person on the bus. (*Authority*)
		[A target] cursed at [his/her] grandfather. (*Subversion*)
	Competition (Positive/Negative)	[A target] worked hard to best [his/her] rivals. (*Positive Competition*)
		[A target] spreads bad rumors about [his/her] rival to win the student council election. (*Negative Competition*)
	Social-oriented (Altruism/Selfishness)	[A target] donated a large amount of money to unfortunate neighbors, although [he/she] lives hand-to-mouth. (*Altruism*)
		[A target] never participated in the group project but still received a good grade for the group work. (*Selfishness*)
	General social affairs (Positive/Negative)	[A target] baked cookies with [his/her] neighbor. (*Positive General Social Affairs*)
		[A target] went to see a movie alone because [he/she] could not find anyone who wanted to join. (*Negative General social Affairs*)

For scenario *content*, we attempted to capture social information via the events of an individual (the target) over a range of categories that spanned from more to less significant events; we did this with eight content domains constructed as dimensions, with each domain having negative and positive counterparts. Five of the eight sociality dimensions were adapted from the moral foundations theory [76–79]: harm, fairness, community, authority, purity. In our study, we used the following: pro-vs-anti-social, shortened to *prosociality* (*care/harm*), *fairness* (*fair/cheating*), *community* (*group loyalty/betrayal*), *respect* (*authority/subversion*), and *purity* (*sanctity/degradation*). The sixth was a content domain for *competition* (both *positive* and *negative*), with the intention of especially comparing competition to *respect* (*authority/subversion*), with both related to relative social value and position in society. The seventh and eighth social categories were *social-vs-self-oriented*, shortened to *social-oriented* (*altruism/selfishness*), and *general social affairs* (*positive/negative*)—with both intended to be progressively less impactful than the moral dimensions. For example, for *social-oriented* (*altruism/selfishness*), although selfishness is generally viewed as a negative characteristic, it is often more tolerable than more egregious offenses. In addition, for *altruism* here, we focus on something more akin to reciprocal altruism, or at least with a clearer possibility (and consideration) of receiving something of value in return (e.g., repayment or reputation gain); this is as opposed to *care* under prosociality, where we focused more on heroic-type acts, without any concern for payback. For *general social affairs*, this content domain was designed to test the significance of everyday events in people’s lives.

After an experimenter (JL) developed the scenarios, two other colleagues (including JK) examined every scenario to verify and thus approve or reject each one. There were 48 separate gossip categories (3 target identity * 2 valence * 8 content), and each category was replicated three times. As a result, 144 different gossip scenarios were used in the experiment. Examples are shown in Table 4.

### Ratings

On every trial, we asked participants to rate each event scenario (on a scale of -3 to +3) according to four different categories to determine how the scenarios were actually received with respect to (1) subjective valence, (2) ordinariness, (3) interest level, and (4) emotion. Subject valence ratings enabled us to validate the valence categories. Ordinariness examined whether the rarity of the scenario regardless of event specifics drove gossiping. The final two were used to gauge how invested participants were in the scenarios—did they take the events seriously as good examples of real life. We first asked them to rate their interest level for each scenario, with the hypothesis that if it were interesting to them, those spread would be rated higher. We also asked the even stronger question of whether the event scenarios actually elicited emotion, with the hypothesis that, if so, those rated more highly for emotion would be spread most.

### Procedure

Participants first completed a general survey upon arrival to the laboratory, which included the acquisition of participants’ background information including their name, age, level of education, and importantly, five names of their close friends. The five names were then immediately inserted into the experimental task as the names of ingroup members (discussed in the Scenarios section above). The participants were then given the instructions about the experimental procedure. The term “gossip” was avoided while the experimenter was explaining the brief purpose and the task steps to participants. Words such as “social information,” “given sentences,” or “scenarios” were used instead.

**Fig 3:**
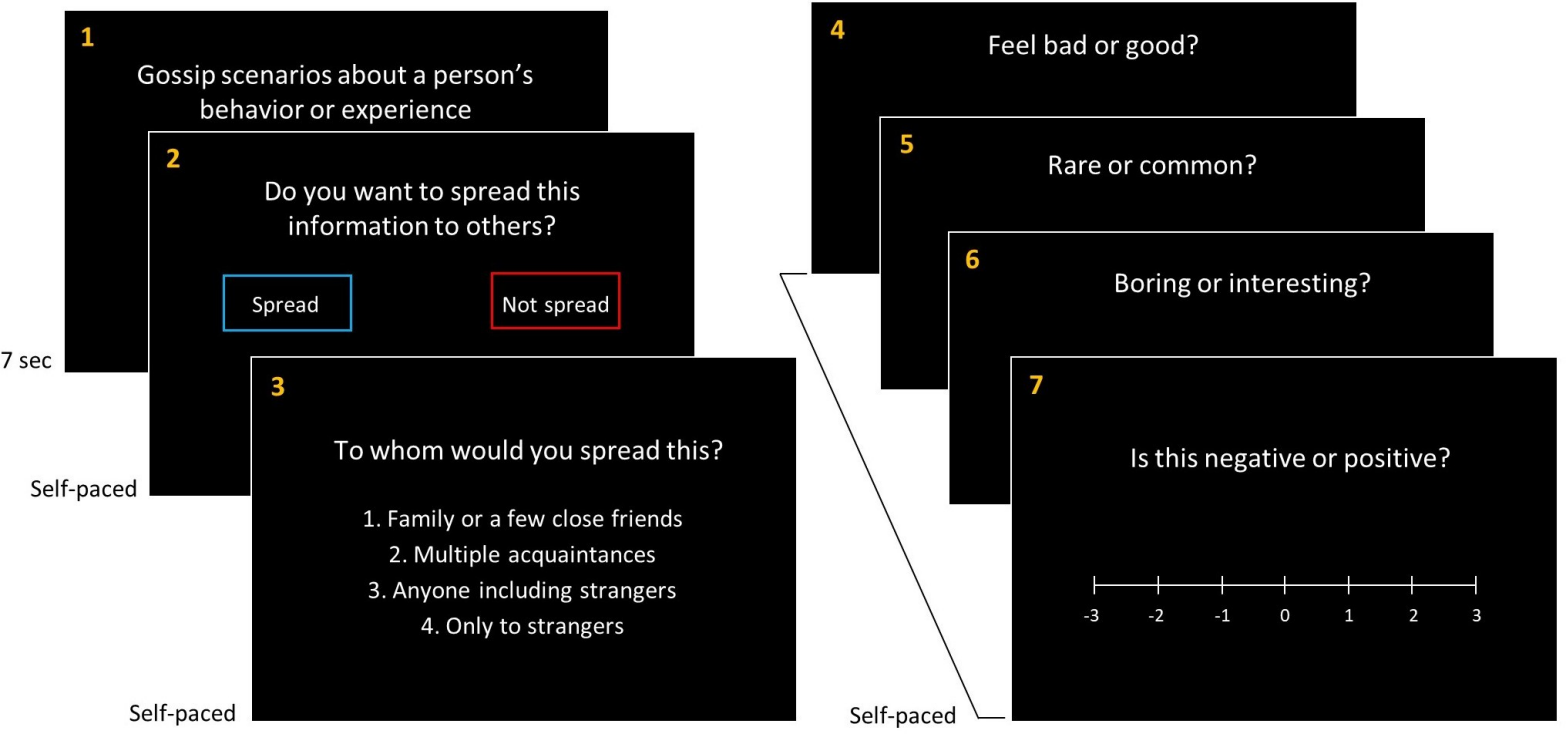
Experimental procedures. The experimental task involved a total of seven screen displays on a computer monitor: (1) a gossip scenario was shown for seven seconds; (2) participants were then asked to decide whether or not to spread the given information to other people by pressing buttons on a keyboard; (3) if they chose to spread the gossip, they were given four options of gossip receivers: family members or a few close friends, multiple acquaintances, anyone including a total stranger, and strangers only, with receiver type to be examined in a future article; and (4)-(7) the participants were then asked to rate each scenario (from -3 to +3) on emotion (4), ordinariness (5), interest level (6), and subjective valence (7).

The experimental task involved a total of seven screen displays on a computer monitor. First, a gossip scenario was shown for seven seconds (Fig 3). After reading the scenario, participants were asked to decide whether or not to spread the given information to other people by pressing buttons on a keyboard. If they chose to spread the gossip, they were given four options of gossip receivers: (1) family members or a few close friends, (2) multiple acquaintances, (3) anyone including a total stranger, and (4) strangers only. The current paper focused on the effects of target, content, and valence on the first question of whether people would spread the given information, with receiver type to be examined in a future article. If participants chose not to spread the gossip, this question regarding the types of gossip receivers was skipped. These first three steps of the task were designed to mimic real social decision-making with respect to gossiping behavior as closely as possible. The monitor displaying various social scenarios served as an initial piece of gossip, and thus each participant played a role as a gossip receiver who could then either become a gossiper (by spreading given scenarios) or not (by choosing not to spread given scenarios). Our interest was in examining the participant in the role as a possible gossiper.

After deciding whether or not to spread any given scenario, the participants were then asked to rate each scenario (from -3 to +3) on the four categories in the following order: (1) emotion, (2) ordinariness, (3) interest level, and (4) subjective valence (Fig 3). There was no time limit for these ratings. Prior to the actual task sessions, a short trial session was conducted to acquaint participants with the procedure and verify that they clearly understood every step, and had no questions after the orientation session. To avoid any confusion, terms used in the sessions, such as emotion, commonness, interest level, and valence were clearly defined. The entire experimental session (including completing the survey) took approximately 80–90 minutes to complete.

### Statistical analysis

The data were analyzed using SPSS 22.0 software (SPSS Inc., Chicago, IL) and MATLAB R2014b (MathWorks, Nattick, MA). The effects of repeated variables (target identity, content, and valence) on mean spread rates of the scenarios were analyzed using repeated measures analysis of variance (ANOVA). As stated, the between-subjects results will be reported in a subsequent paper. After performing a repeated measures ANOVA, the mean of each variable was compared using two-tailed student’s t-tests for further analysis of variable interaction, with Bonferroni correction for multiple comparisons. T-tests were also used to examine the participants’ behavioral responses on the four additional ratings: emotion, ordinariness, interest, and rightness of each scenario.

## Results

### Participant ratings

Before considering the three independent variables (target, content, valence), we first examined the ratings by the participants to determine how the scenarios were actually received. First, we examined subjective valence to verify that our valence categories aligned with those of the subjects. As can be seen in S1 Fig in S1 Text, they indeed experienced the positive scenarios as positive (mean = 1.37, SEM = 0.015) and the negative ones as negative (mean = -1.67, SEM = 0.013). Next, to gauge how invested participants were in the scenarios, we compared the gossiped scenarios versus those not gossiped in terms of subjective valence (‘how good’ or ‘how bad’), interest level, and emotion, with the hypotheses that stronger valenced (‘better’ or worse’), more interesting, and more emotionally evocative scenarios would be gossiped more. In all cases, this is what we found, for both positive (subjective valence: t_7340_ = -33.24, p < 0.001; interest: t_7142_ = -55.90, p < 0.001; emotion: t_6721_ = -39.95) and negative scenarios (subjective valence: t_7258_ = 24.78, p < 0.001; interest: t_7101_ = -35.97, p < 0.001; emotion: t_7324_ = 22.63, p < 0.001) (S1A-S1F Fig in S1 Text). Thus, the scenarios were indeed meaningful to the participants, to the point of eliciting emotion (*feeling* good or bad). We next asked whether the gossiped scenarios tended to be events out of the ordinary (rare vs. common), and they were, for both positive (t_7305_ = 23.95, p < 0.001) and negative (t_7329_ = 9.81, p < 0.001) scenarios (S1G & S1H Fig in S1 Text). Thus, the gossiped scenarios were more highly valenced, interesting, emotionally evocative, and rare.

Of the scenarios gossiped, when comparing positive versus negative scenarios directly, we found that (a) both tended to be comparably rarer events (with no significant difference between them for commonness) (S2A Fig in S1 Text), (b) the positive scenarios gossiped were significantly more interesting than the negative ones (t_7279_ = -11.54, p < 0.001) (S2B Fig in S1 Text), while (c) the negative scenarios were more emotionally evocative (t_7305_ = -6.49, p < 0.001) (S2C Fig in S1 Text). Overall, then, both the positive and negative scenarios engaged the participants significantly on all ratings measures, with evidence that the positive ones gossiped were particularly more interesting, and the negative ones particularly emotion inducing. S3 Fig in S1 Text shows all participant ratings findings in radial graphs. We next examine the three main independent variables (target, valence, content) and their interactions.

### Overall

Overall, there was a significant main effect for all three independent variables: (1) target (F_2_ = 200.84, p < 0.001, η_p_^2^ = 0.077); (2) valence (F_1_ = 6.85, p < 0.01, η_p_^2^ = 0.001); and (3) content (F_7_ = 134.01, p < 0.001, η_p_^2^ = 0.162). Moreover, all three two-way interactions were significant: (1) target-by-valence (F_2_ = 38.25, p < 0.001, η_p_^2^ = 0.016); (2) target-by-content (F_14_ = 26.88, p < 0.001, η_p_^2^ = 0.072); and (3) content-by-valence (F_7_ = 27.61, p < 0.001, η_p_^2^ = 0.038). Finally, the three-way interaction was also significant: target-by-content-by-valence (F_14_ = 7.52, p < 0.001, η_p_^2^ = 0.021). We next examine these results more closely, together with the follow-up *post hoc* analyses and implications.

### Target

**Fig 4:**
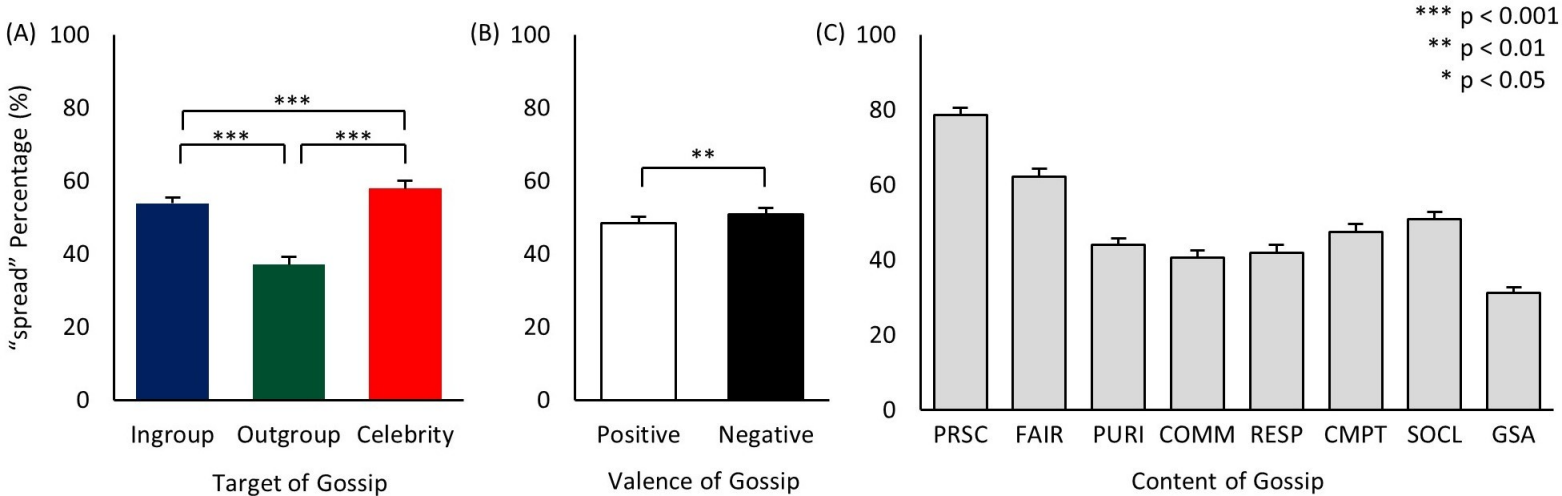
Mean spread rates of target, valence, and content of gossip (i.e., main effects). (A) Mean spread rates of ingroup, outgroup, and celebrity gossip. (B) Mean spread rates of positive and negative gossip. (C) Mean spread rates of eight different contents of gossip (see S1 Table in S1 Text for statistical significance): PRSC (prosociality), FAIR (fairness), PURI (purity), COMM (community), RESP (respect), CMPT (competition), SOCL (social-oriented), GSA (general social affairs). ** p < 0.01, *** p < 0.001.

As seen in Fig 4A, the predicted relations “Celebrity > Ingroup >> Outgroup” of *Hypothesis 1* were observed. First, gossip about outgroup targets was spread significantly less than gossip about the other two groups, and thus as expected the behavior of outgroup members generated less desire to communicate to others. As evaluated via Table 2, the underlying causes of this lack of spreading information about them likely stems from low interest, intimacy, and expected status [4].

In addition, celebrity gossip was spread significantly more than ingroup gossip. The higher spreading rate of celebrity gossip could reflect heightened gossiping of this target group, especially due to their higher profile and status; yet it can also reflect reduced gossiping of ingroup targets (e.g., potentially avoiding talking about own ingroup members due to the potential costs involved). Both do appear to be the case under closer examination of the results below.

### Valence

For valence, we found that, overall, negative information was slightly (but significantly) more gossiped than positive information (Fig 4B), generally supporting *Hypothesis 2 ‘Negative > Positive >> 0*’. Valence (positive/negative) of transmitted information has been shown to influence the gossiper’s decision, with valence in fact being included in some definitions as a necessary component of gossip [9, 11]. Indeed, the popular view appears to assume that gossiping mostly involves negatively valenced events (pejorative, salacious), and there certainly is evidence for it [16, 83, 85, 86]. And yet others have also found evidence that positively valenced events (affirmatory, curious, information gathering) are also gossiped [8, 32, 57, 59–61].

In our framework, because both positive and negative information can provide benefits to knowing and transmitting it, both were expected to be significantly spread. Our results thus show that the potential value of positive gossip does not appear to be neglected by people. At the same time, negative content was nonetheless more significantly spread than positive. The reasons for negative gossiping could vary. First, negatively valenced events may simply elicit more interest by virtue of being more unexpected, less common, if people are generally expected to behave properly; however, we did not find such a difference in participant ratings of commonness (see *Participant Ratings* above). Yet the negative scenarios gossiped did elicit more emotion than the positive ones (S2C Fig in S1 Text). It would thus appear that the emotion underpinned taking actions, suggesting that there is purpose behind negative gossiping, such as to punish wrongful behavior or to inform receivers of possible problematic interactions with the target in the future. This would indeed be expected from an evolutionary perspective.

### Target x valence

**Fig 5:**
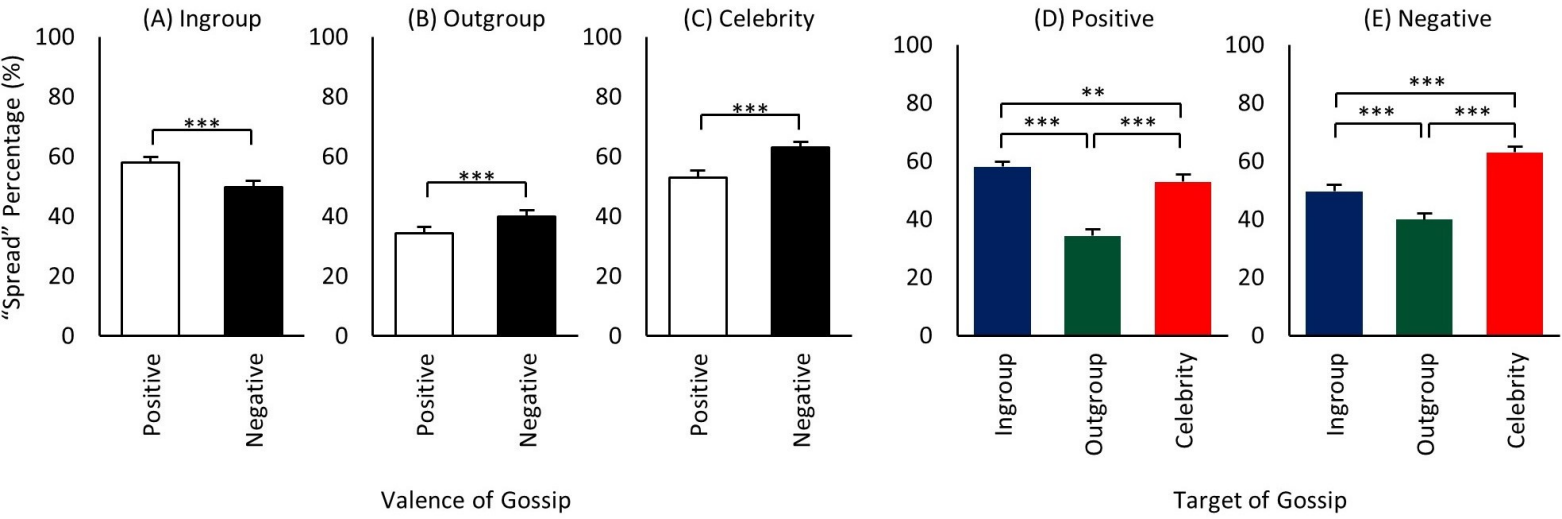
Mean gossip spread rates of 2-way interaction ‘target x valence’. (A) Mean spread rates of positive and negative gossip of ingroup target. (B) Mean spread rates of positive and negative gossip of outgroup target. (C) Mean spread rates of positive and negative gossip of celebrity target. (D) Mean spread rates of positive ingroup, outgroup, and celebrity gossip. (E) Mean spread rates of negative ingroup, outgroup, and celebrity gossip. ** p < 0.01, *** p < 0.001.

The valence main effect showed skewing toward negative gossip (Fig 4B), and we next asked whether this result is generally the same for each target group. That is, what is the breakdown of positive vs. negative gossiping for ingroup, outgroup, and celebrities? There were two main findings. First, in line with the valence main effect, both celebrities and outgroup members yielded more negative than positive spreading (Fig 5B & 5C), supporting *Hypothesis 4a* ‘*Celebrity-Negative > Celebrity-Positive*’, as well as showing the same pattern for outgroup members. Because the costs of spreading information about them is not expected to be high (for either positive or negative), this relation for both celebrities and outgroup would be expected. Moreover, as seen in Fig 5E, the high negative spreading for celebrities in particular further suggests that influence and social status of targets play a significant role in driving people to spread information about them [49, 62, 90].

Second, however, the ingroup result exhibited the opposite pattern: a skewing toward positivity (Fig 5A), as *Hypothesis 4b ‘Ingroup-Positive > Ingroup-Negative’* predicted, and others have found [83, 90, 91]. This result suggests that the ramifications from negative information spreading about ingroup members likely reduces it. (At the same time, it remains possible that the higher positive spreading leads to more direct payoff to the spreader as well.) Finally, the significant differences in Fig 5D and 5E show that the differences seen within the target groups (Fig 5A–5C) extend across them, such that positive spreading is highest for ingroup (Fig 5D), and negative spreading highest for celebrity (Fig 5E). Thus, overall, we found that ‘who the person is’ and ‘content valence’ interact, with positive gossip more dominant when about more intimate ingroup members, and negative gossip for those with whom one does not have to worry about future repercussions, and especially for more influential targets such as celebrities, whom we appear to hold particularly accountable.

### Content

Along with an event scenario’s target and valence, the third critical feature we examined was content–i.e., the topic of the event. S1 Table in S1 Text lists all post-hoc comparison results for content. As seen in Fig 4C (and S1 Table in S1 Text), overall, scenarios involving all five morality dimensions were spread more than the general social affairs content domain, thus supporting *Hypothesis 3a ‘Morality > General Social Affairs’*. But more specifically, even with morality, certain dimensions may be more impactful than others, and this is what we found: *prosociality* and *fairness* were spread the most, followed by the others (*competition*, *purity*, *respect*, and *community*), supporting *Hypothesis 3b* ‘*Prosociality > Fairness > [The others] > General Social Affairs’*. In fact, the results extend beyond our hypotheses by showing more detailed differences among the domains (see S1 Table in S1 Text), which can be more closely examined in future studies.

The results for *content* overall support previous findings that moral issues promote gossip, and perhaps even more so when pitted against other content domains [25, 35–42, 64–66, 68]; and our results extend previous findings to further dimensions of morality and social interaction (although we found qualifying evidence below that context also matters). In particular, our results bear out that *prosociality (care/harm)* and *fairness (fair/cheating)* are especially important, driving higher amounts of spreading. These specific findings make sense with issues of harm and cheating, the respective negative moral components, but may be less clear with the positive counterparts (care and being fair). These issues are examined more closely next.

### Content x valence

**Fig 6:**
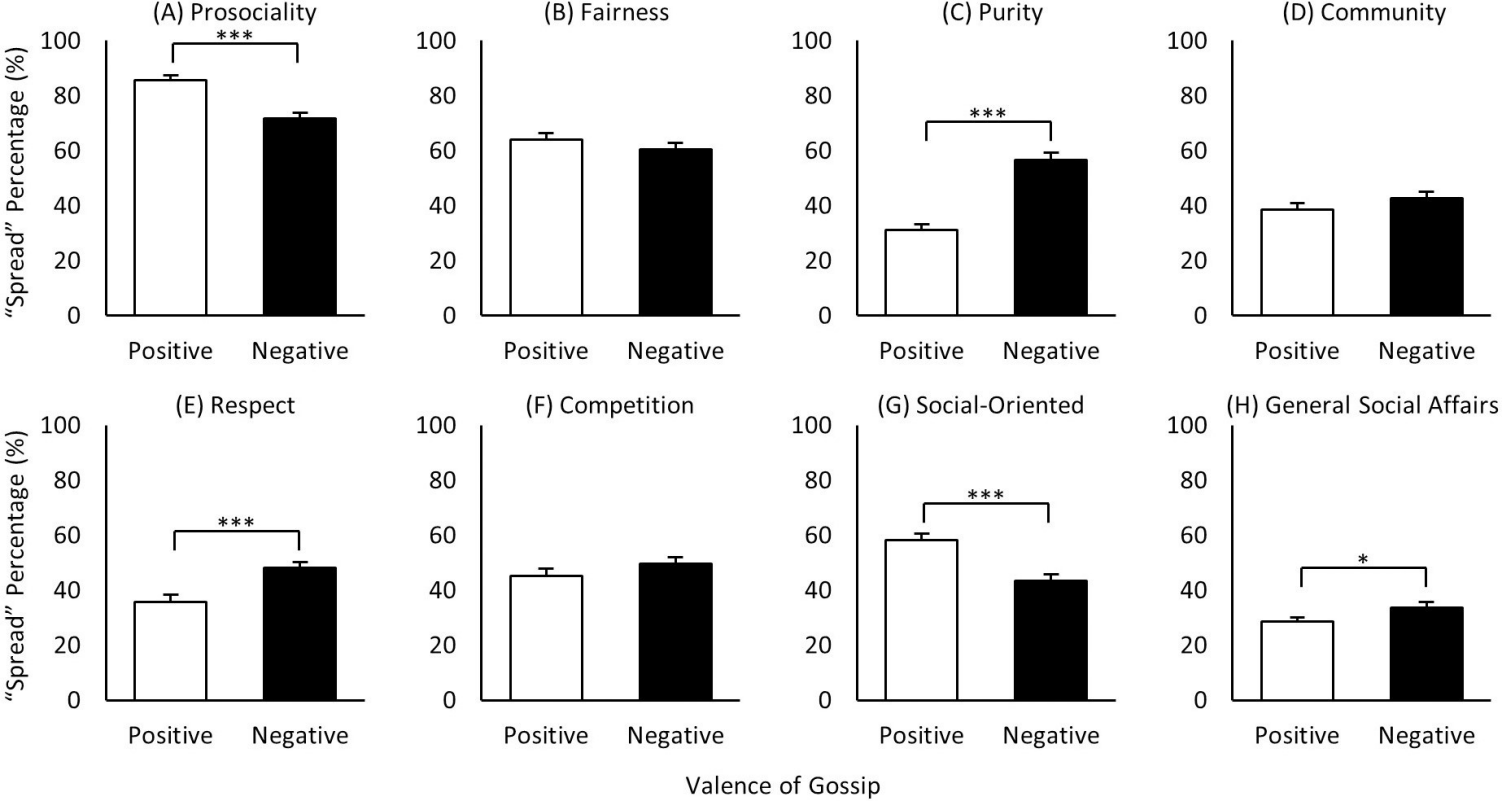
Mean gossip spread rates of 2-way interaction ‘content x valence’. (A)-(H) Mean spread rates of positive and negative gossip for eight different content domains: prosociality (care/harm), fairness (fair/cheating), purity (sanctity/degradation), community (group loyalty/betrayal), respect (authority/subversion), competition (positive/negative), social-oriented (altruism/selfishness), and general social affairs (positive/negative). White bars represent positive and black bars represent negative counterparts of each content domain. * p < 0.05, *** p < 0.001.

Of the eight content domains, three generated more negative spreading: *purity*, *respect*, and *general social affairs* (Fig 6C, 6E & 6H); two generated more positive spreading: *prosociality* and *social-oriented* (Fig 6A & 6G); and three were more comparable for both positive and negative: *fairness*, *community*, and *competition* (Fig 6B, 6D & 6F). Thus, for the two content domains that consistently generated the most gossip, *prosociality* and *fairness* (Fig 4C), neither case exhibited more negative than positive gossip, which *Hypotheses 6a and b (*‘*Harm > Care >> 0*’ and ‘*Cheating > Fairness >> 0*’) had predicted. Indeed, the opposite was found, with *care* spread significantly more than *harm*, and *fair* more than *cheating*, though not significantly. Similarly, *altruism* was also spread significantly more than *selfishness*. Thus, perhaps surprisingly, but intriguingly, social information spreading does not appear to be dominated by concerns of threat and loss. Rather, we again found evidence that positive gossip appears to offer comparable value–or even greater in some cases–to the gossiper (and others) than does negative gossip, providing additional support for positive information providing value for the gossiper and receivers [32, 60, 61]. In short, these results show that gossiping is not driven mostly by ‘mean-girl’ type motives, with a great deal rather about goodness, valor, and instances of high empathy. Quiet heroism also impresses.

For the three generating more negative gossip, the significance of *subversiveness* and *degradation* (for outgroup too) makes sense since respect for *authority* and *sanctity* (the positive counterparts) may be more generally expected by people, while subversiveness and degradation may be relatively unacceptable. In fact, these results may be even more significant in Confucian-based societies such as Korea, where authority and sanctity are considered particularly important virtues. The greater spreading of the negative component of *general social affairs* may similarly reflect day-to-day social affairs being typically positive and expected as the default (e.g., Person-X baked cookies with their neighbor last weekend), with negative ones (e.g., Person-Y broke up with his girlfriend) in any case requiring additional attention and resolution.

In sum, for five of the eight content domains, positively valenced content was spread equivalently to or even more than negatively valenced. For the three in which negatively valenced dominated (*respect*, *purity*, and *general social affairs*), the positive counterpart might be considered more expected (and thus less gossip worthy), whereas the negative side may be relatively more unexpected and newsworthy. And again, we perhaps surprisingly found that, rather than the need to manage potential threats, positive characteristics of targets and their behavior promote a great deal of information spreading, indicating that gossip is a mechanism to mine value wherever it resides in the social world. Although tests of certain moral dimensions such as *fairness* have been successfully used to uncover fundamental aspects of our cooperative/competitive social behavior in numerous studies [25, 35–42], our results show that the various moral dimensions also differ in important ways, requiring more comprehensive examination of the critical dimensions underlying our social behavior in future studies.

### Content x target

Our findings thus far suggest that, rather than with trivial matters, gossip may be driven more to the extent information transmission will impact the gossiper’s social environment–i.e., based on the actual potential effects it may have. Thus, moral dimensions in particular would be ripe for spreading, and accumulating evidence, together with our content main-effect and interaction-with-valence results bear this out [25, 35–42, 64–66, 68]. Taken together, does this mean that morality-based scenarios always reflect the most potential impact and thus should always lead to greater spreading than other content domains? A more complete assessment of the significance of social knowledge suggests that this may not always be the case. That is, social knowledge is needed not only to maintain social order, but for all aspects of social interaction including in day-to-day activities; and social interactions require ‘mind-reading’ of others to anticipate their thoughts, feelings, intentions, actions, etc. [5, 72, 73, 92, 93]. Thus, any knowledge that can provide accurate models of others’ minds may be highly valued under various circumstances: in other words, context should matter. For example, does gossiping about certain types of content depend on who is being gossiped about (the target)? There were three general findings (with S2 Table in S1 Text listing all post-hoc comparison results).

**Fig 7:**
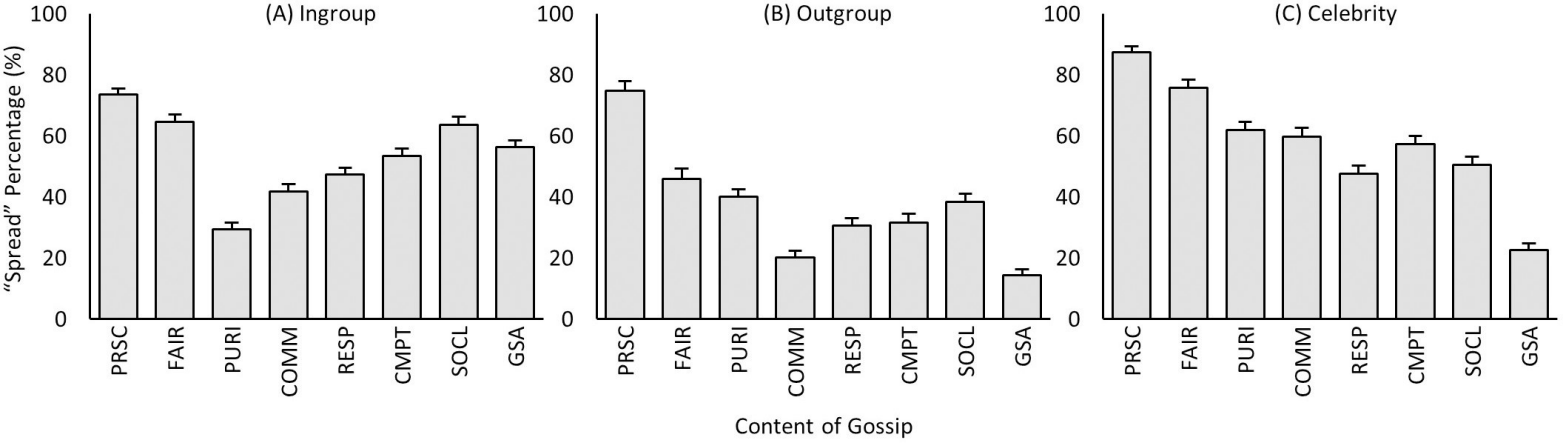
Mean gossip spread rates of 2-way interaction ‘content x target’. (A)-(C) Mean spread rates of eight different content domains for ingroup, Outgroup, and celebrity target, respectively. (PRSC: prosociality, FAIR: fairness, PURI: purity, COMM: community, RESP: respect, CMPT: competition, SOCL: social-oriented, GSA: general social affairs.) See S2 Table in S1 Text for statistical significance.

**Fig 8:**
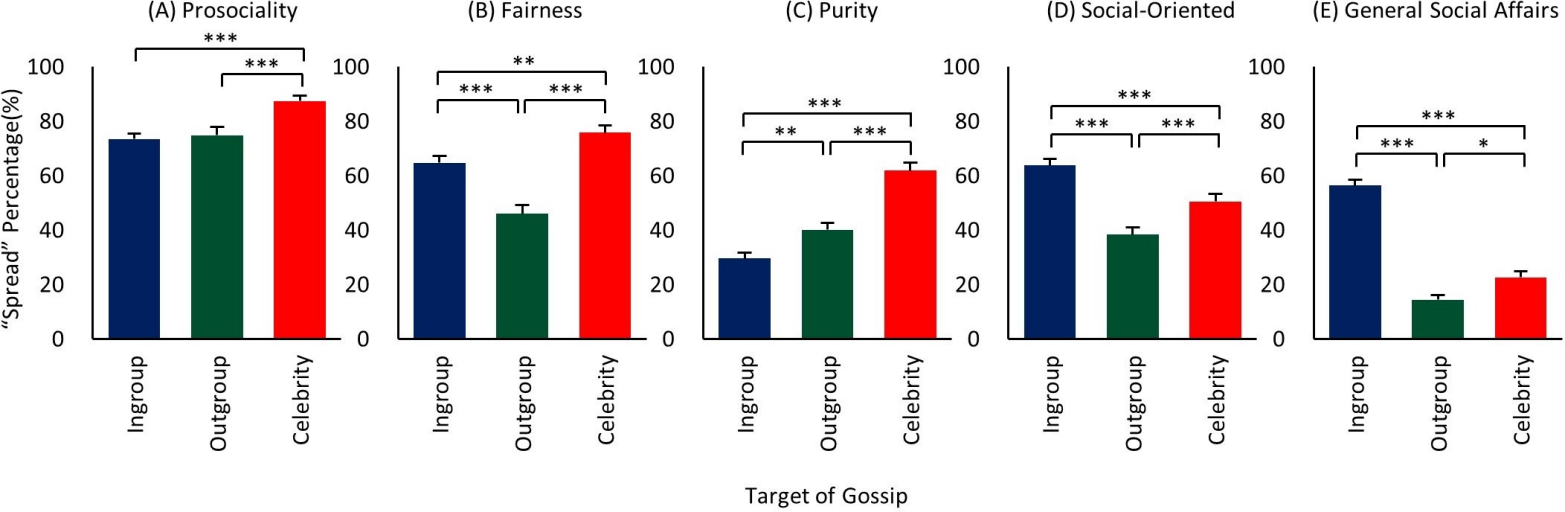
Mean gossip spread rates of 2-way interaction ‘content x target’. (A)-(C) Mean spread rate of ingroup, outgroup, and celebrity gossip for social-oriented, general social affairs, and purity, respectively. * p < 0.05, ** p < 0.01, *** p < 0.001.

First, as seen in Fig 7A–7C (and S2 Table in S1 Text), for all three target groups, *prosociality* and *fairness* were spread the most (reflecting the content main effect shown in Fig 4C and supporting *Hypothesis 3*). Thus, these two categories transcended target group specifics: they mattered ‘no matter what’. Nonetheless, we did find differences among the target groups. Fig 8A shows for *prosociality*, spreading occurred thus: ‘celebrity > ingroup ≈ outgroup’, partially supporting Hypothesis 5a (*Celebrity-Prosociality > Ingroup-Prosociality >> Outgroup-Prosociality*) with celebrity spreading higher than ingroup spreading, though with ingroup and outgroup being comparable. This result appears to attest to the significance of *prosociality* (*care/harm*) not only for maintaining proper status standing with the most influential targets, but for everyone. In addition, as seen in Fig 8B, we found evidence supporting Hypothesis 5b (*Celebrity-Fairness > Ingroup-Fairness >> Outgroup-Fairness*). And yet, as seen in Fig 7B, fairness was the second most spread content for outgroup–again generally attesting to the significance of this morality dimension.

For the second main finding, more general, everyday sociality, as represented by *social-oriented* and *general social affairs*, was more relevant for ingroup targets (Fig 8D & 8E), not only supporting Hypothesis 5c (*Ingroup-General-Social-Affairs > Celebrity-Same >> Outgroup-Same*), but going beyond to include *social-oriented*. For *social-oriented*, it appears that even smaller examples of good or bad behavior (i.e., those more typically seen day-to-day with theoretically lower overall impact) are worthy of notice and bringing to the attention of others. In fact, these cases of good/bad social behavior may reflect greater consequences for ingroup members. That is, altruistic behavior involves an action prioritizing one’s group (or society) over self. Because celebrities are more distant from us, *altruistic* gossip may not have as great an impact on the potential gossiper as ingroup altruistic gossip, having more of an indirect and thus muted influence. This same logic would apply with *selfishness* gossip. Potential free-riding among our ingroup members (rather than celebrities) may more directly and thus significantly affect our lives.

The heightened spreading of *general social affairs* gossip (including a target’s personal traits, experience, activities, etc.) is particularly telling. Even though such information may not on the surface appear impactful, our theoretical framework highlights the fact that in order to predict other’s behavior accurately, we must attempt to read their minds, which requires a detailed model of their current knowledge, goals, etc. [5, 72, 73, 92, 93]. And yet reading minds accurately requires reasonably elaborate models of others’ minds, which can quickly become computationally challenging as the number of individuals expands. To manage this challenge, people do not generally develop elaborate models of all individuals; rather, they do so with those most relevant to their lives, such as close ingroup members. And to maintain an accurate model of them, we must constantly update it based on events that occur in their lives. Thus, for those closer to us, apparently superficial or mundane events take on greater significance if they affect the accuracy of our mental models of them. This theoretical prediction–*Hypothesis 5c –*is thus supported by the ingroup-target results.

The third main finding was a conspicuous lack of spreading of gossip about *purity* of ingroup members compared to the other target groups (Fig 8C). For *purity*, it is possible that this may be expected from ingroup members and thus not interesting news, but this would seem so for other target groups (i.e., outgroup and celebrities) that showed relatively high *purity* spreading (*purity* was the third most spread content for both celebrities and outgroup; Fig 7B & 7C; S2 Table in S1 Text). Rather, it appears that *purity* as a content type is being actively avoided with ingroup targets and perhaps generally a taboo subject. Indeed, topics about personal hygiene and sexual affairs may be related closely to the social reputation of a target, and therefore especially risky to spread for ingroup targets with high intimacy.

In sum, we did find that morality issues appear particularly important with respect to status and influence (reflected in celebrity information most spread), and we nonetheless also found them to be particularly important in general, especially *prosociality* and *fairness*, attesting to the importance of the potential impact on future social interactions: gossip is generally not trivial. At the same time, we also found that other types of social information remain meaningful, even for the most apparently mundane topics of *general social affairs*, attesting to the significance of context, and more specifically, to the importance of maintaining accurate models of others’ minds, at least of those closest to you. Finally, we once again found that morality itself must now be understood in terms of specific dimensions, given that the results can vary quite widely based on the specific dimension considered (as especially occurred with *purity* in our case).

### Content x valence x target

**Fig 9:**
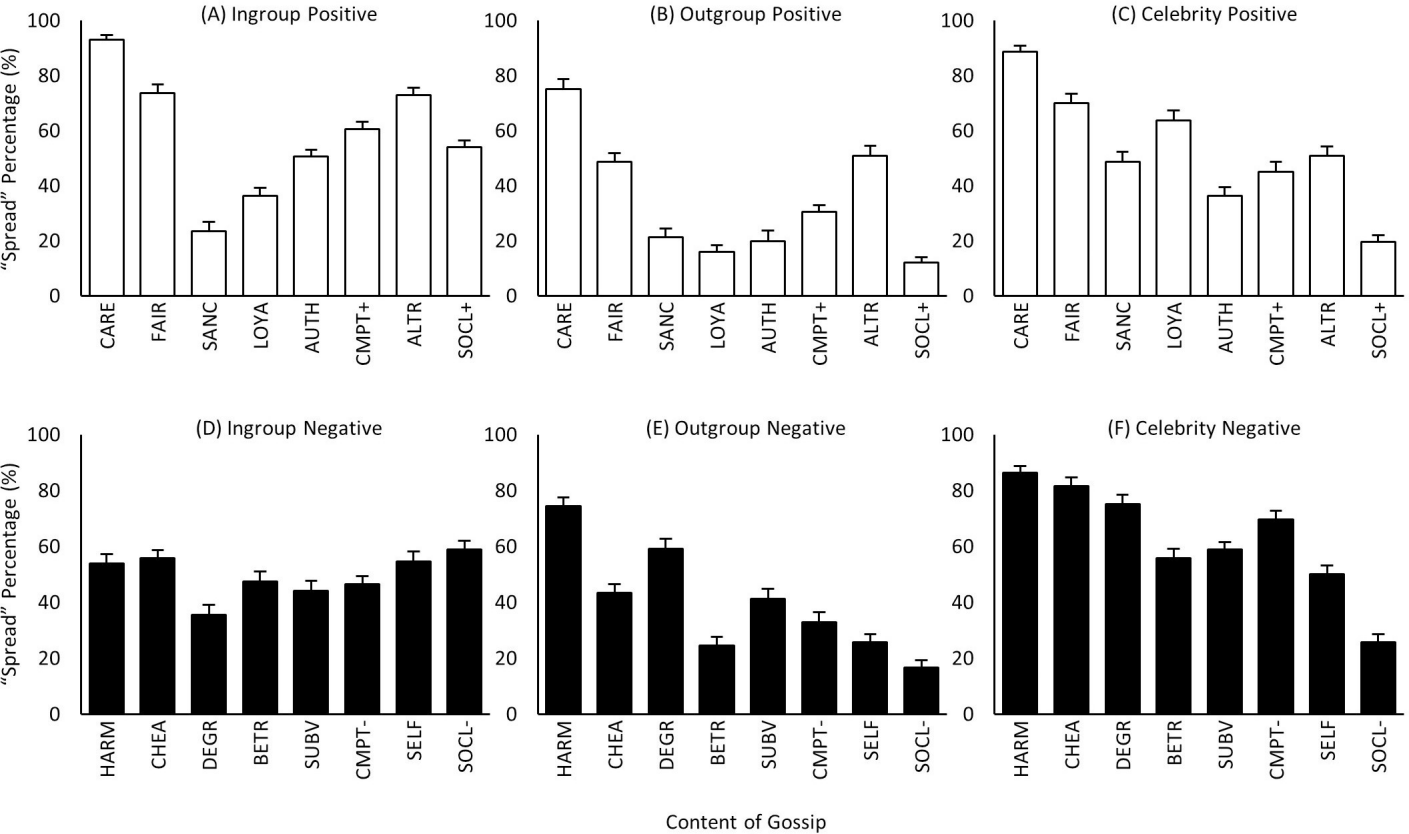
Mean gossip spread rates of 3-way interaction ‘content x valence x target’. (A)-(C) Mean spread rates of content domains for positive gossip of ingroup, outgroup, and celebrity targets, respectively. (D)-(F) Mean spread rates of content domains for negative gossip of ingroup, outgroup, and celebrity targets, respectively. (CARE: care, FAIR: fairness, SANC: sanctity, LOYA: group loyalty, AUTH: authority, CMPT+: positive competition, ALTR: altruism, SOCL+: positive general social affairs for positive, and HARM: harm, CHEA: cheating, DEGR: degradation, BETR: betrayal, SUBV: subversion, CMPT-: negative competition, SELF: selfishness, SOCL-: negative general social affairs for negative counterparts of 8 contents.) See S3 Table in S1 Text for statistical significance.

We next asked how valence factors into these ‘content x target’ findings: for example, is there strong interest in both positive and negative aspects of the content dimensions or does it skew more negative with celebrities, positive with ingroup? Fig 9 shows the spread rates of content domains for positive (A-C) and negative (D-F) gossip of ingroup, outgroup, and celebrity targets (with S3 Table in S1 Text listing all post-hoc comparison results). We examine each target group in turn.

**Fig 10:**
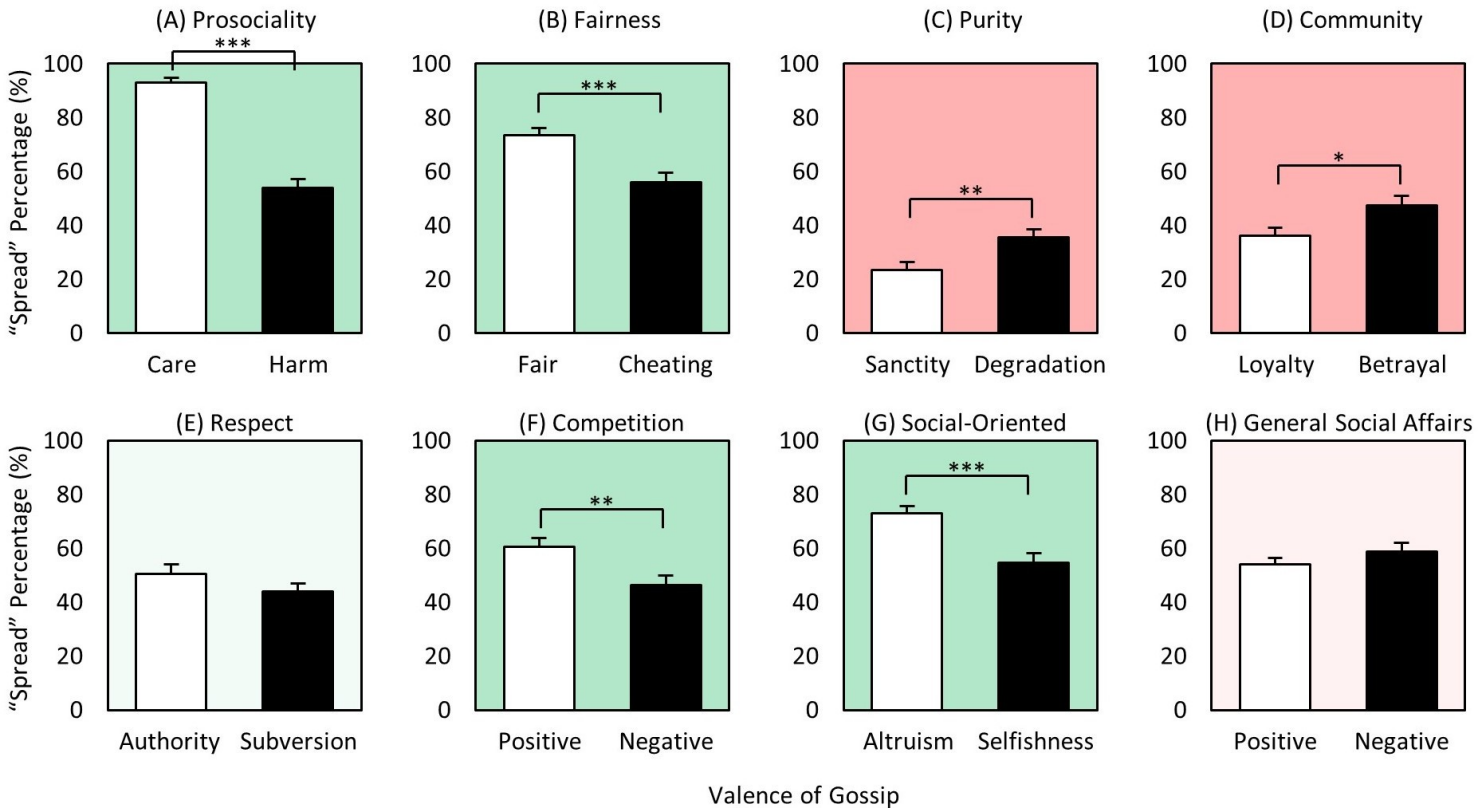
Mean gossip spread rates about ingroup targets for positive versus negative events. The graphs with green shade ((A), (B), (F) & (G)) are those with higher positive spreading rates than negative spreading rates. The graphs with red shade ((C) & (D)) are those with the opposite pattern. The graphs with pale green (E) and pale red (H) are not statistically significant yet show the trends of the graphs with the corresponding colors. * p < 0.05, ** p < 0.01, *** p < 0.001.

Fig 10 shows the gossip spread rates about *ingroup* targets for positive versus negative events for each content domain. The most dominant finding was ingroup targets skewing positive, with five of the eight (63%) content domains having higher positive than negative spreading: four significantly (*care/harm*, *fair/cheating*, *positive/negative competition*, and *altruism/selfishness*) (Fig 10A, 10B, 10F & 10G), and one (*authority/subversion*) trending the same direction (Fig 10E). The other three domains showed higher negative than positive spreading: two significantly (*group loyalty/betrayal*, *sanctity/degradation*) (Fig 10C & 10D), and one trending so (*general social affairs*) (Fig 10H). For the latter, perhaps because day-to-day social events have weaker cost to spread regardless of valence, people spread them almost equally. The *sanctity/degradation* result (Figs 9A & 9D and 10C) again points to this domain as being particularly charged, and for ingroup targets, likely involving privacy and potential shaming concerns. At the same time, *sanctity* cases (“Sarah always washes her hand carefully before she leaves washroom.”) could potentially entail more expected events, less worthy of spreading. Lowered spreading of *group loyalty* cases (“Friend showed patriotism in a room full of people from various country.”) may also be more expected for ingroup targets, with close friends already evidencing loyalty (Figs 9A and 10D).

In *Supporting Information*, we consider these findings in more detail, focusing on potential evidence for whether skewing positive for ingroup targets results from more positive gossiping or less negative gossiping (i.e., elevated positivity or avoided negativity). In fact, further examination of the negative spreading of ingroup targets suggests that there may be ceiling (i.e., do not want to be too negative, no matter what the content) and floor (i.e., but yet some negativity necessary for all content types) effects on negative gossiping: resulting in a relatively flat line for negative spreading across content types, at ~50% spreading rate (Fig 9D; S3 Table in S1 Text). In *Supporting Information*, we also address the questions of why certain topics were avoided, and why there was a general negativity-avoidance effect for ingroup targets.

Taken together, the ingroup target findings indicate that multiple dimensions induce significant positive gossiping of ingroup members (with *sanctity* and to some extent *group loyalty* as exceptions), highlighting the significance and value of positive information about ingroup target members to people. At the same time, multiple factors appear to dampen negative gossiping of ingroup members, likely including potential repercussions, other means to communicate to the target, and perhaps more leeway with and empathy toward equal and equivalently lower status individuals. Further empirical and computational research can help to delineate the potential influence of such factors.

**Fig 11:**
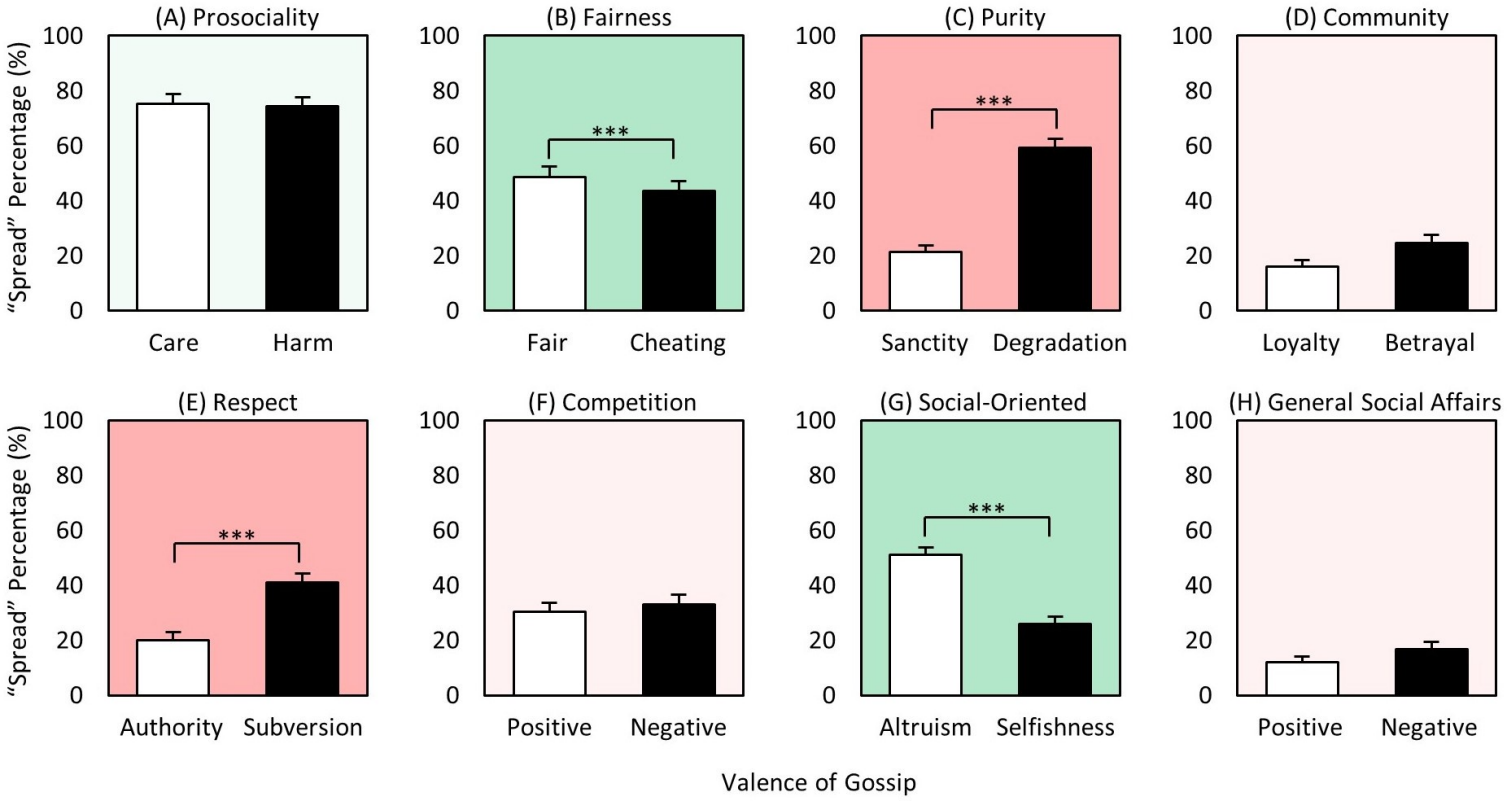
Mean gossip spread rates about outgroup targets for positive versus negative events. The graphs with green shade ((B) & (G)) are those with higher positive spreading rates than negative spreading rates. The graphs with red shade ((C) & (E)) are those with the opposite pattern. The graphs with pale green (A) and pale red ((D), (F) & (H)) are not statistically significant yet show the trends of the graphs with the corresponding colors. * p < 0.05, ** p < 0.01, *** p < 0.001.

Fig 11 shows the gossip spread rates about *outgroup* targets for positive versus negative events for each content domain. As suggested in the target by valence results (Fig 5B), spreading of outgroup target information, although generally lower, skewed negative, with five of the eight (63%) content domains having higher negative than positive spreading: though only two significantly (*sanctity/degradation*, *authority/subversion*) (Fig 11C & 11E), and the other three (*group loyalty/betrayal*, *positive/negative competition*, *positive/negative general social affairs*) trending the same direction (Fig 11D, 11F & 11H). The other three domains showed higher positive than negative spreading: with two significantly so (*fairness/cheating*, *altruism/selfishness*) (Fig 11B & 11G), and one trending (*care/harm*) (Fig 11A). As seen especially in Fig 9B & 9E (as well as Fig 11), for outgroup targets, the most striking negative events were *harm* and *degradation*, followed by *cheating* and *subversion*. Thus, these topics draw significant interest and concern for any person, independent of who they are, how close they are to you, and their social status. For positive events, the ones that particularly draw spreading even about strangers are *care*, *fairness*, and *altruistic* behavior. Thus, being a good, caring, selfless person to others appears to universally draw interest and a desire to tell others.

**Fig 12:**
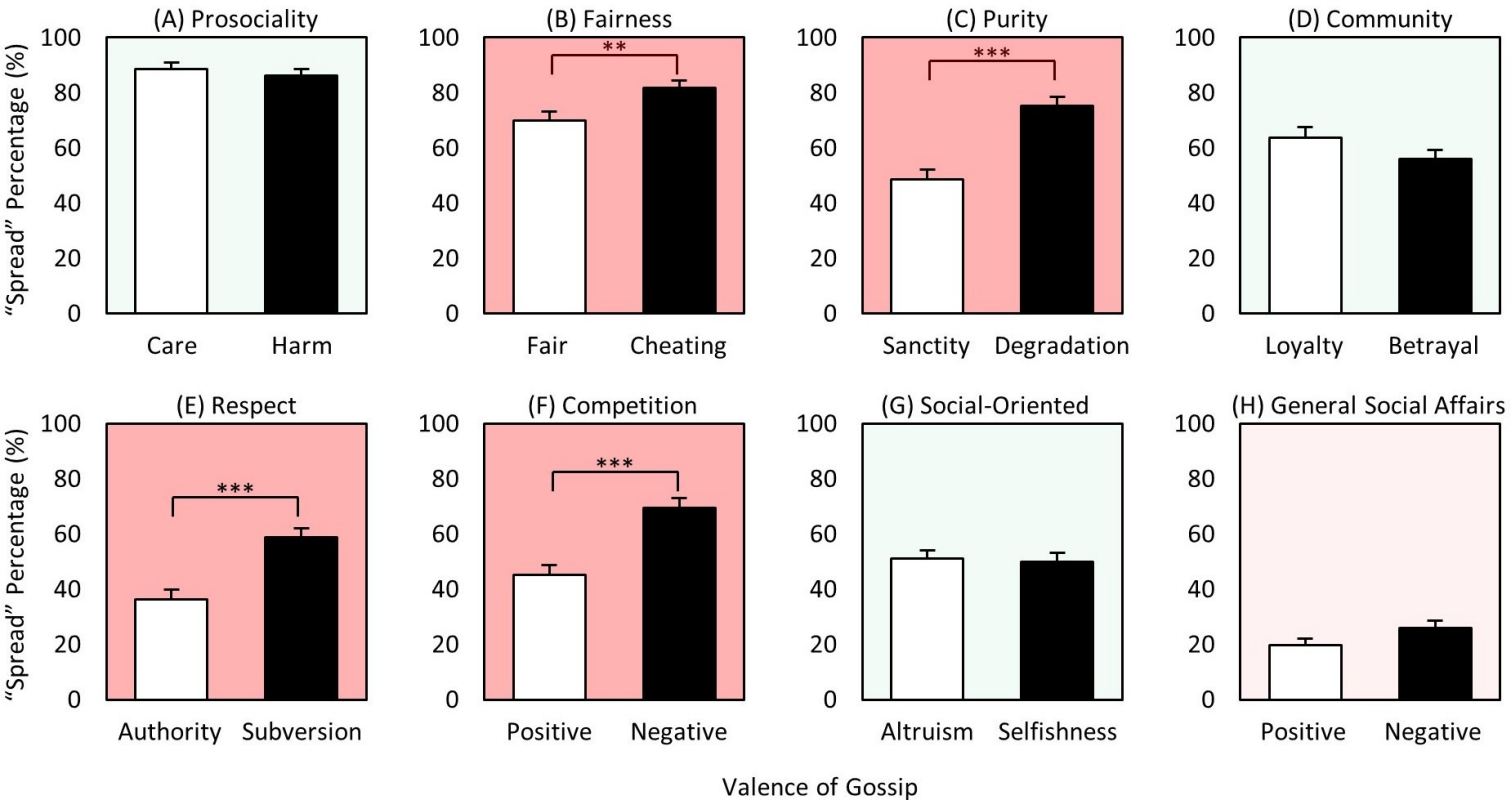
Mean gossip spread rates about celebrity targets for positive versus negative events. The graphs with red shade ((B), (C), (E) & (F)) are those with higher positive spreading rates than negative spreading rates. The graph with pale red (H) is not statistically significant yet has the trend of the graphs with its corresponding color. The graphs with pale green ((A) & (G)) are not statistically significant yet show the opposite trend of the graphs with red shade. * p < 0.05, ** p < 0.01, *** p < 0.001.

For celebrities, Fig 12 shows the gossip spread rates for positive versus negative events for each content domain. As anticipated by the target by valence results (Fig 5C), gossiping about celebrities generally skewed negative, with higher negativity rates for five of the eight (63%) content domains compared to their positive valence counterpart: significantly so for four (*fair/cheating*, *sanctity/degradation*, *authority/subversion*, *positive/negative competition*) (Fig 12B, 12C, 12E & 12F), and trending for the fifth (*general social affairs*), with the latter likely reflecting a generally lower interest in this content domain for celebrities (for both positive and negative events) (Figs 12H and 9C & 9F). Although popular sentiment might suspect more negative gossiping about celebrity targets, it is nonetheless decidedly moral-based, and significantly skewed to negative behavior overall (toward others, society, health), showing that those holding higher social status and outsized interest are indeed held to high standards of behavior (whether directly spread for this reason or for any other, such as entertainment from scandalous behavior that nonetheless leads to reputation effects) (see *Supporting Information* for further examination of status effects) [49, 62, 90].

For celebrity and outgroup targets, *degradation* appears to be especially upsetting, leading to a high rate of gossiping about it (Figs 9E & 9F, 11C, 12C; S3 Table in S1 Text). This finding supports others that show the strong reactions people have to basic biological matters related to health, wellbeing, and sexuality (such as the relationship between *purity* and disgust in [94, 95]). Which is all the more intriguing that *purity* (*sanctity/degradation*) is generally depressed for ingroup targets, as discussed under *Content x Target*, in which for ingroup it is quite clearly inhibited and the least spread content domain, further suggesting the charged and particularly personal nature of this information (Fig 9D).

At the same time, for celebrities, three of the content domains (*care/harm*, *altruism/selfishness*, *group loyalty/betrayal*) showed higher spreading of positive vs. negative events, though none of these were significant (Fig 10A, 10D & 10G)–and thus, generally showing comparable spreading of the two valences. In fact, as seen especially in Fig 9C, *caring*, *fair*, and *altruistic* positive acts were all fairly highly spread for celebrity targets–just as with outgroup targets–again pointing to a potential universal interest in and appreciation for good, caring, and selfless acts toward others. Kindness indeed gets people’s attention. Finally, the spread rate for *group loyalty* with celebrity targets also appeared particularly high (Fig 9C)–even higher than the other target groups (Fig 9A & 9B), which we revisit in the Discussion below.

In sum, the three-way interaction results attest to the complexity of human social interactions in general, and social information communication more specifically. Context clearly matters. At the same time, general patterns were again observed, such as positive skewing for ingroup, negative for celebrities. Even so, exceptions existed, with, for example, lowered positive spreading about ingroup targets for domains potentially more expected (*group loyalty*) or more damaging (*purity* in general), and higher positive spreading about celebrity and outgroup targets for acts of kindness.

## Discussion

In this study we sought to test the extent to which gossiping behavior could be understood using a model of social information communication [72, 73]. This model is not unlike others, investigating the fundamental motives behind gossip behaviors [e.g., 20, 24, 26, 28]; however, our model extends the existing ones, providing a more comprehensive sociocognitive-neuroeconomic account of social information communication that more closely models the human mind/brain, allowing us to understand and explain the findings under one consistent and detailed framework. To test the six model predictions, we first asked participants to read various gossip scenarios (e.g., Person-X cheated on the final exam), and then asked whether they would gossip this information to others. The scenarios varied the target person that the gossip is about (ingroup, celebrities, or outgroup), valence (whether positive or negative), and content (eight different domains).

### Main findings

The *target* main effect (celebrities > ingroup >> outgroup) provides evidence for *interest* more generally [4, 9, 14–19], and relationship *intimacy* [2, 23, 34, 49–53] and social *influence* [24, 25, 27, 35–48] more specifically as important factors driving people to gossip. Since intimacy or closeness implies having more meaning and influence in an individual’s day-to-day life, and influence or status implies social attention, power, and influence, both factors support the notion that functional significance strongly drives gossiping.

For *valence*, we found negative events to be spread more than positive ones. Indeed, the functional role of negative gossiping–e.g., to punish the target, protect receivers, and potentially promote oneself [24, 25, 27, 35–48, 83, 90, 96]–appeared prominent and clear, especially given that we did not find evidence for negative gossiping being based simply on its intrigue or entertainment value (with ratings for ‘rarity’ comparable to positive scenarios and lower than positive scenarios for ‘interest level’).

Although, overall, negative valence promoted gossiping more than positively valenced scenarios, a persistent finding across the study nevertheless was that positively valenced scenarios perhaps rather surprisingly promoted gossip to a large degree: overall (i.e., not greatly lower than negative: 48.41% spreading for positive vs. 50.76% for negative), being higher than negative spreading for ingroup (target x valence), and being comparable to or even higher than negative spreading for specific content dimensions, even for celebrities and outgroup [61]. For example, the positive gossiping rate was especially high for *care*, *fairness*, and *altruism* for all target groups, indicating that these positive acts resonate perhaps universally: such kindness and regard for others permeates the social network. Our results (including the ratings scores for subjective valence, interest and emotion) thus join the others that have found positive events to also strongly drive gossip [8, 32, 57, 59–61]. Moreover, given that this occurred with all content domains (i.e., all showed significant positive gossiping), the results suggest that along with correction and ‘punishment’, positive feedback and information sharing comparably influence social behavior–not only for motives such as social bonding, but also for social control via strengthening and promoting it [26]. This is especially suggested given the high rates of positive gossiping across the moral domains. Indeed, rather surprisingly, we even found prosociality to produce more positive (*care*) than negative (*harm*) gossiping.

The results for *content* overall supported our hypothesis that, in general, moral dimensions would be spread most, and in particular, that *prosociality* (*care/harm*) and *fairness* (*fairness/cheating*) would be most spread. These align with previous findings that highlight the functional significance of gossip as it relates to cooperation, competition, and other moral dimensions, and we extend the findings to further dimensions of morality [25, 35–42, 64–66, 68]. Thus, almost diametrically opposed to the presumed trivial nature of gossip, we found the most impactful moral dimensions to be most spread, attesting to the importance of gossip on the regulation of societal members and the influence on future social interactions.

At the same time, scenarios representing all of the moral domains (and all content domains for that matter) were significantly spread at various rates, indicating the need to study these various domains and their differences more closely, with dominant paradigms that have focused on cooperation and competition, for example, useful in their own right, but not well representing other important social dimensions. For instance, the results for *purity* were particularly interesting, revealing that, on the one hand, the dimension appears to be universally meaningful (here: across all target groups), and yet, on the other hand, treated exceptionally, at least for perhaps particular targets (here: ingroup) and cultures (here: Korean). Indeed, gossip spreading about targets’ norm violation has been shown to depend on cultural context [63, 69]. Future research is therefore needed to clarify how and why individuals and societies respond the way they do to cases involving purity (e.g., gossiping vs. other means of communication, or perhaps even suppression)–and, in fact, for all individual dimensions of social interaction (i.e., moral and others). To be sure, our findings also point again to the significance of the relationships and interdependencies among the gossip parties themselves (i.e., gossiper-receiver, gossiper-target, and receiver-target) [20]. Moreover, we believe these questions are particularly ripe for additional modeling [97–100] and neural imaging studies [101–103] to help clarify the factors, their relationships, and the underlying mechanisms that drive the sociocognitive-neuroeconomic decisions involving social interaction and communication.

In addition to moral dimensions, we nonetheless also found that other types of knowledge may be highly valued under various circumstances; in other words, context is critical: for example, with more personal and day-to-day sociality (i.e., *general social affairs* and *social-oriented*) being more important with ingroup targets. Because this more seemingly mundane social information was differentially important for ingroup members, it suggests that its significance derives from the desire to be updated about the basic activities of those close to you [28]. From a sociocognitive perspective, it implies the need to maintain accurate knowledge of them: i.e., an accurate model of their minds, including their current knowledge, interests, intentions, activities, etc. [5, 72, 73, 92, 93]. Moreover, in doing this, a sense of solidarity and feelings of community among ingroup members also develops [23, 34, 46, 49–53].

A dominant finding in the two-way (*target x valence*) and three-way (*content x valence x target*) interactions was that spreading information about ingroup targets skewed positive, suggesting either that positive events were more meaningful and thus being spread more for ingroup targets [83, 90], or spreading negative information about them might be costlier [20]–and we found evidence for both–especially the latter. That is, for spreading negative information about ingroup members, the overall results suggest a general *negativity-avoidance effect* for ingroup targets. This result is in line with previous findings that people perceive a gossiper who shares favorable and therefore positive information about others more positively, which should be especially important among ingroup members [65, 104–107].

Multiple factors likely dampen negative gossiping of ingroup members, including potential repercussions, other means to communicate to the target, and perhaps more leeway with and empathy toward equal and equivalently lower status individuals [20, 21, 49, 108, 109]. Further empirical and computational research can help to delineate the potential influence of such factors on negative gossiping about ingroup members (including both the ‘ceiling’ and ‘floor’ components). In any case, this avoidance of negativity with ingroup members provides evidence for our predictions (*Hypothesis 4b*), and more specifically, that the *net effect of the overall expected outcome* influences the gossiper’s decision to gossip [20, 57].

Another dominant finding was that spreading information about celebrities generally skewed negative. This overall effect is likely at least partially due to the lowered risk of repercussions to the gossiper; but the evidence further suggests that the *social status* of celebrities is a major driving factor underlying the heightened negative gossiping, with the intention of lowering that of the celebrity (and thereby raising the gossiper’s own status, and perhaps the receivers’ as well), at least among those within the gossiper’s purview. This finding thus supports others showing that in an environment where vertical hierarchy exists, people with low status tend to gossip about high-status individuals with more power [28, 49]. Gossiping negatively about people with power (especially about moral contents) has been called a *weapons-of-the-weak* mechanism [110] or subordinate strategy [see 111] whereby low-status, relatively powerless individuals use gossip as a weapon to pressure more privileged individuals [26]. An examination of the results for the specific content domains further showed that for higher-status individuals (celebrities), *loyalty* and *humility* warrants action (i.e., gossiping), as do cases of trying to beat others in underhanded ways or otherwise cheating the system [83].

In contrast, the greater interest in *altruism* (over *selfishness*) and *fairness* (over *cheating*) for both ingroup and outgroup targets suggests that such selfless and fair acts are especially impressive when conducted by those of lesser status and means. Moreover, *authority* and *positive competition* with ingroup targets appear to generate action (receiving higher gossiping rates), suggesting that these topics are more relevant among relatively lower (compared to celebrities) and more equal status ingroup members, with *positive competition* suggesting scenarios of ambition or achievement resonate more. At the same time, selfish behavior of ingroup and outgroup people are relatively less spread, with such acts appearing to be more tolerated in those with less means and status. In sum, our results support our hypotheses and others findings that status is a major factor determining the extent of and types of gossip [28, 49]. Further empirical and computational work can extend our findings by delineating exactly how status interacts with other factors to promote social interactions, such as with social information spreading [e.g., 112]. In any case, the evidence for status considerations again attests to the importance of functional value driving gossiping behavior.

For outgroup targets, we generally found a relative lack of interest, with many results significantly weaker compared to ingroup and celebrities, and thus supporting our hypotheses (especially *Hypothesis 1*). Even this result may be a bit surprising if one generally construes the “outgroup” as outsiders, and thus potential threats, enemies, etc. However, comparable to the findings of others, we found that the usual use of the “outgroup” concept requires a more nuanced appreciation [113]. Those considered as viable threats likely evoke sufficient interest that warrants action, rather than generally being ignored. Yet it is also unfortunately probable that a relative lack of empathy can be seen with strangers in general, making it more difficult to care sufficiently in their affairs [114, 115].

At the same time, however, even for outgroup members (i.e., complete strangers), some content domains were generally important in our study, including a heightened rate of information spreading for both *care* and *harm* (i.e., the *prosociality* domain), *fair* and *cheating* (i.e., the *fairness* domain), and *altruism* and *selfishness* (i.e., the *social-oriented* domain). This heightened spreading for all three target groups indicates a strong interest in *prosociality*, *fairness*, and *social-orientation* that is worthy of disseminating to others regardless of the actor involved–i.e., prominent universals for all members of society. These results are in line with others that show that stories of strangers can also elicit interest and therefore produce gossip if the events can offer useful life lessons and strategies [4, 28].

### Study limitations

There are some study limitations that should be considered. First, we did not test our hypotheses in a more natural context where gossip triads are interacting freely in spontaneous situations. The clear advantage of field studies using various methods such as eavesdropping [6, 8, 116], daily diary surveys [117], and experience sampling methods [59] is that a potentially rich set of observational data that reflects real-life gossiping behavior can be collected [see 59, 96]. And there are indeed cases where behavior observed in the laboratory may not appear outside it [118, 119], requiring all laboratory studies to consider the ecological validity. Here, we took several steps to minimize the gap between the natural and laboratory settings. First, we note that much of the information people learn about others (including ingroup members) these days comes from texts read on electronic devices (phones, computers). Second, prior to the experiment we asked participants to submit the names of their closest friends (i.e., ingroup) so that the ingroup scenarios would feel like actually receiving information about them. We also chose well-known Korean celebrities, and used foreign names as strangers for the outgroup. Third, we also gave participants thorough instruction to assume that every piece of information provided during the task is real; and we received several comments during the post-experiment verbal interview directly stating that the scenarios felt realistic, even being “shocked” by some of the extreme cases (e.g., with harm or degradation contents). Fourth, and importantly, our experiment was also designed to address ecological validity directly by asking participants to rate each scenario according to subjective valence, ordinariness, interest level, and emotion (after they chose whether to gossip or not). The scenarios participants chose to gossip were considered to have higher valence (whether positive or negative), to be rarer, more interesting, and more emotionally evocative—thus showing that they were meaningful to the participants. Fifth, and finally, the fact that many of our results match those of other studies helps to support the validity of our experimental paradigm.

Indeed, experimental paradigms such as ours help complement others by enabling clearer and more precise testing of relevant factors. Nonetheless, it is clear that future research is needed to further validate our findings, not only with methodologies described above, but others as well, such as curating and examining natural interactions on social media (such as Facebook or Twitter).

A second limitation is that the behavioral-based study is nevertheless limited in the precision it can achieve, not enabling tests of more detailed processes in our theoretical framework. Future research can hopefully utilize our framework and test paradigm to examine, for example, the neural processes underlying the gossip decision (such as the benefits vs. costs computations in the brain that drive gossiping). A few studies have investigated neural activity during gossip [101–103], but many unknown factors remain. Third, it is clear that many more factors remain to be examined, such as how the choice of *receiver* influences the gossip decision, as the possible gossiper may keep quiet [20] or selectively expose the target information [49, 120] depending on the receiver’s identity. Other factors include more specific detail about the target and event content, especially to better characterize the more nuanced context effects.

## Conclusions

In sum, we found that, rather than being superficial, merely entertaining, or driven by reasons of malicious intent, social information spreading–gossip–reflects a key mechanism by which social interaction, cohesion, and order take place in a large social network [2, 3, 23–25, 34–42, 49–54]. In addition, the current study successfully categorized contents of gossip into eight categories, elaborating the meaning of “norm” with seven of them, beyond the predominant focus on cooperation in general. As a result, we showed that various critical types of content produce different rates of gossip, suggesting that each gossip content evokes different reasons to gossip and carries different values based on its relative benefits and costs [e.g., 20, 27, 45, 57]. At the same time, our study was able to examine critical target effects, specifically influence, closeness, and interest, on gossip behavior, supporting and extending previous findings on, for example, celebrities alone [28]; or in more limited settings, such as game-theory scenarios [35–42], workplace, and schools [20, 24, 48–50, 52, 57, 60, 83, 84, 90, 120]; or those requiring further validation under more controlled conditions [59, 96]. Furthermore, our study was able to examine this wide range of factors with both positive and negative valence cases, enabling clearer comparison of the influence of each (supporting, for example, the significance of negative events, and yet as well a high prevalence of positive gossiping, especially for ingroup members) [8, 11, 16, 61, 83, 85, 86, 90].

Our model based on the human higher mind/brain was able to successfully predict *a priori* how these multiple factors related to the target (i.e., who they are) and the content of the event (i.e., what they did) influence gossiping. To our knowledge, no one has heretofore combined all of these factors, produced *a priori* predictions about them, and provided direct evidence to test them. Our results further suggest that the functional value derived from gossiping involves the highest levels of our sociocognitive abilities, such as the knowledge and processing required to maintain and continuously update models of others’ minds, use it to predict their behavior, and perform and maintain the sophisticated social accounting necessary for successful and valuable long-term social interactions [1, 72–75, 121, 122]. We hope our paradigm and findings can provide specific hypotheses for future examination of the underlying neural mechanisms. Indeed, because of the myriad factors involved as people pursue goals within a complex multi-agent world, theoretical, computational and empirical analyses are needed to delineate exactly how we succeed under such challenging conditions–and choose not to live as an island.

## Supporting information

S1 Text Supporting information of paper: Supplementary figures, tables, and text. (PDF)

S1 Dataset Experimental data. (XLSX)
