# Supplementary material for: How ‘who someone is’ and ‘what they did’ influences gossiping about them
Source: PLoS One. 2022 Jul 6;17(7):e0269812. doi: 10.1371/journal.pone.0269812 (PMC9258836; doi:10.1371/journal.pone.0269812)
Supplement: S1 Text — (PDF) [file pone.0269812.s001.pdf]

S1 Supporting information of paper: Supplementary figures, tables, and text

1 Supplementary Figures

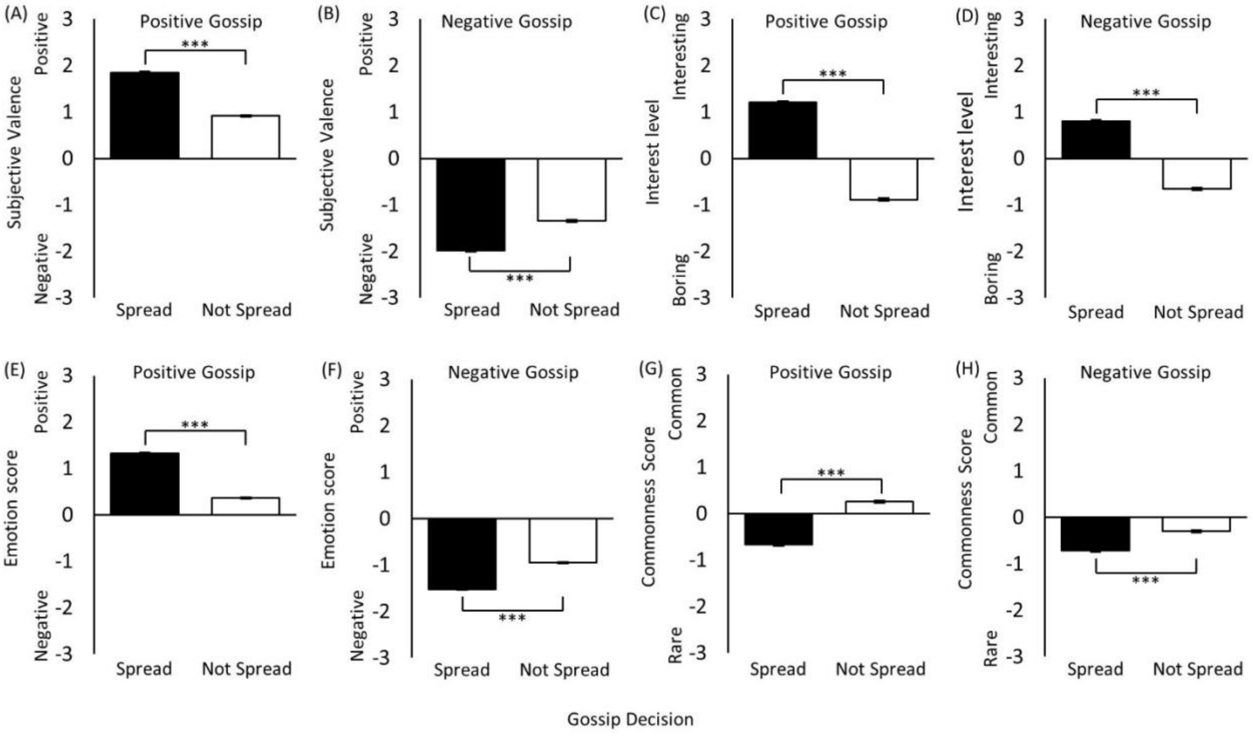

Fig S1. Mean scores of the gossip scenarios that were spread and not spread. (A) & (B) Results of the subjective valence scale for both positive and negative valenced gossip: from -3 (the event described on the screen is very negative/wrong/bad) to 0 (neutral) to 3 (very positive/right/good). (C) & (D) Results of the interest level scale for positive and negative gossip: from -3 (very boring) to 0 (neutral) to 3 (very interesting). (E) & (F) Results of the emotion scale for positive and negative gossip: from -3 (evoked very negative emotion) to 0 (neutral emotion) to 3 (very positive emotion). (G) & (H) Results of the commonness scale for positive and negative gossip: from -3 (very rare) to 0 (neither rare nor common) to 3 (very common). *** $p < 0.001$.

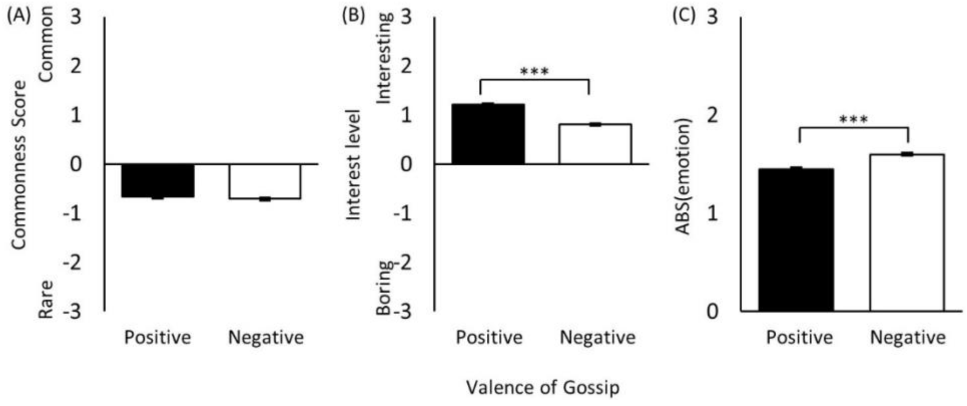

Fig S2. Mean scores of positive and negative gossip scenarios that were spread. (A) & (B) Mean scores of commonness and interest level rated by the participants. Scores of gossip scenarios that were "spread" were calculated. (C) The absolute values of the mean scores on emotion scale. *** $p < 0.001$.

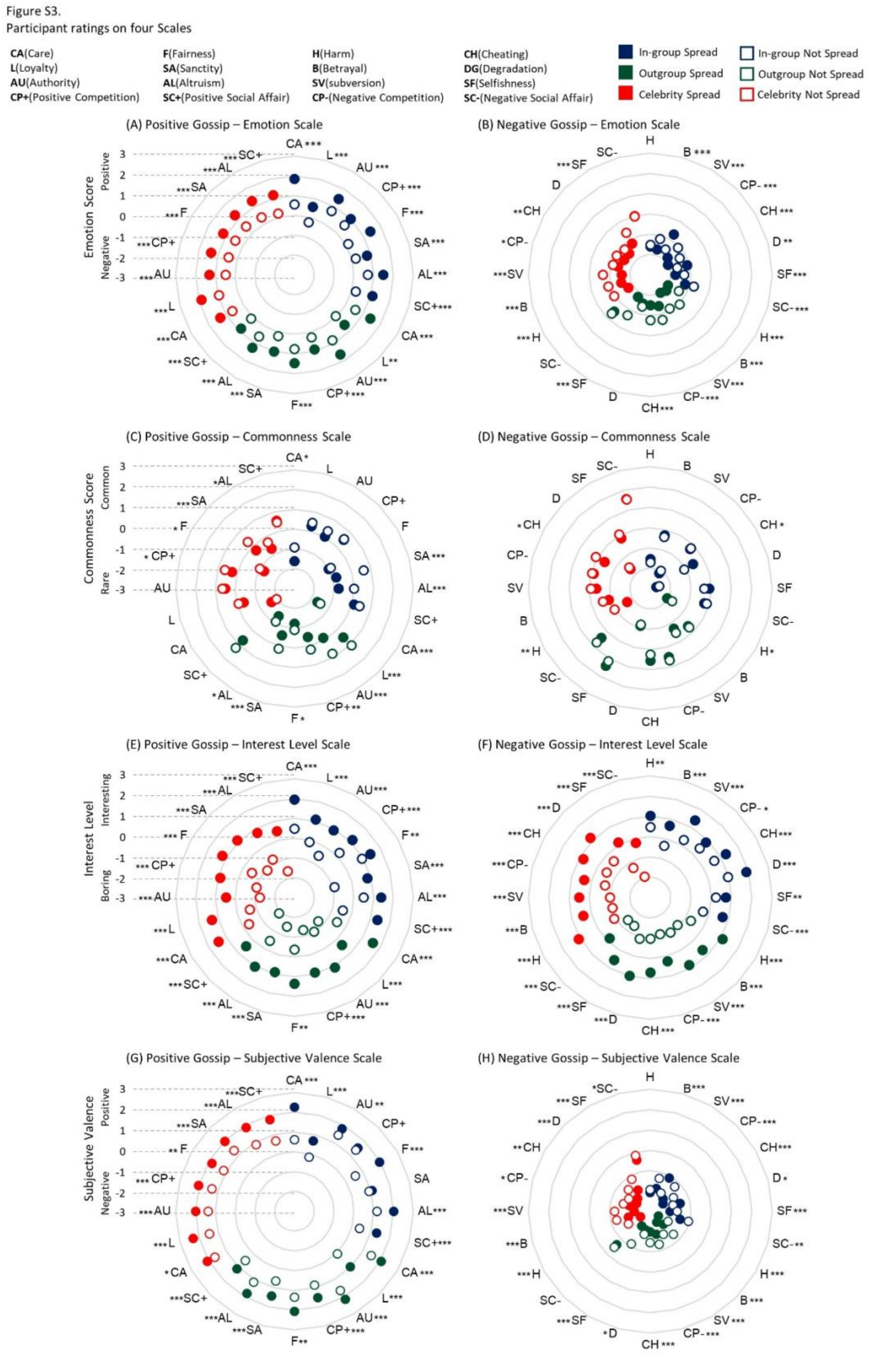

Figure S3. Mean scores difference between the gossip scenarios that were spread and not spread. (A) & (B) Results of the emotion scale for positive and negative gossip: from -3 (very

negative emotion) to 0 (neutral emotion) to 3 (very positive emotion). (C) & (D) Results of the commonness scale for positive and negative gossip: from -3 (very rare) to 0 (neither rare nor common) to 3 (very common). (E) & (F) Results of the interest level scale for positive and negative gossip: from -3 (very boring) to 0 (neutral) to 3 (very interesting). (G) & (H) Results of the subjective valence scale for positive and negative gossip: from -3 (very negative/wrong/bad) to 0 (neutral) to 3 (very positive/right/good).

2 Supplementary Tables

Table S1. Post-hoc comparisons for content main effect (Bonferroni-corrected for multiple comparisons). * p < 0.05, ** p < 0.01, *** p < 0.001.

Contents	*Contents*	*t-value*	*p-value*	*95% Confidence interval for difference*	
				Lower bound	*Upper bound*
Prosociality (care/harm)	Community (loyalty/betrayal)	21.116	0.000***	0.323	0.436
	Respect (authority/subversion)	20.449	0.000***	0.311	0.424
	Competition (positive/negative)	17.281	0.000***	0.255	0.367
	Fairness (fair/cheating)	9.113	0.000***	0.108	0.220
	Purity (sanctity/degradation)	19.282	0.000***	0.291	0.403
	Social-oriented (altruism/selfishness)	15.392	0.000***	0.221	0.333
	General social affairs (positive/negative)	26.339	0.000***	0.418	0.531
Community (loyalty/betrayal)	Prosociality (care/harm)	-21.116	0.000***	-0.436	-0.323
	Respect (authority/subversion)	-0.666	1.000	-0.068	0.044
	Competition (positive/negative)	-3.834	0.004**	-0.125	-0.012
	Fairness (fair/cheating)	-12.003	0.000***	-0.272	-0.159
	Purity (sanctity/degradation)	-1.816	1.000	-0.089	0.024

	Social-oriented (altruism/selfishness)	-5.668	0.000***	-0.159	-0.046
	General social affairs (positive/negative)	5.279	0.000***	0.039	0.151
Respect (authority/subversion)	Prosociality (care/harm)	-20.449	0.000***	-0.424	-0.311
	Community (loyalty/betrayal)	0.666	1.000	-0.044	0.068
	Competition (positive/negative)	-3.167	0.046*	-0.113	0.000
	Fairness (fair/cheating)	-11.336	0.000***	-0.260	-0.147
	Purity (sanctity/degradation)	-1.150	1.000	-0.077	0.036
	Social-oriented (altruism/selfishness)	-5.001	0.000***	-0.147	-0.034
	General social affairs (positive/negative)	5.946	0.000***	0.051	0.163
Competition (positive/negative)	Prosociality (care/harm)	-17.281	0.000***	-0.367	-0.255
	Community (loyalty/betrayal)	3.834	0.004**	0.012	0.125
	Respect (authority/subversion)	3.167	0.046*	0.000	0.113
	Fairness (fair/cheating)	-8.168	0.000***	-0.203	-0.091
	Purity (sanctity/degradation)	1.998	1.000	-0.020	0.092
	Social-oriented (altruism/selfishness)	-1.876	1.000	-0.090	0.022
	General social affairs (positive/negative)	9.057	0.000***	0.107	0.220

Fairness (fair/cheating)	Prosociality (care/harm)	-9.113	0.000***	-0.220	-0.108
	Community (loyalty/betrayal)	12.003	0.000***	0.159	0.272
	Respect (authority/subversion)	11.336	0.000***	0.147	0.260
	Competition (positive/negative)	8.168	0.000***	0.091	0.203
	Purity (sanctity/degradation)	10.169	0.000***	0.127	0.239
	Social-oriented (altruism/selfishness)	6.279	0.000***	0.057	0.170
	General social affairs (positive/negative)	17.226	0.000***	0.254	0.367
Purity (sanctity/degradation)	Prosociality (care/harm)	-19.282	0.000***	-0.403	-0.291
	Community (loyalty/betrayal)	1.816	1.000	-0.024	0.089
	Respect (authority/subversion)	1.150	1.000	-0.036	0.077
	Competition (positive/negative)	-1.998	1.000	-0.092	0.020
	Fairness (fair/cheating)	-10.169	0.000***	-0.239	-0.127
	Social-oriented (altruism/selfishness)	-3.890	0.003**	-0.126	-0.013
	General social affairs (positive/negative)	7.057	0.000***	0.071	0.184
Social-oriented (altruism/selfishness)	Prosociality (care/harm)	-15.392	0.000***	-0.333	-0.221
	Community (loyalty/betrayal)	5.668	0.000***	0.046	0.159

	Respect (authority/subversion)	5.001	0.000***	0.034	0.147
	Competition (positive/negative)	1.876	1.000	-0.022	0.090
	Fairness (fair/cheating)	-6.279	0.000***	-0.170	-0.057
	Purity (sanctity/degradation)	3.890	0.003**	0.013	0.126
	General social affairs (positive/negative)	10.947	0.000***	0.141	0.253
General social affairs (positive/negative)	Prosociality (care/harm)	-26.339	0.000***	-0.531	-0.418
	Community (loyalty/betrayal)	-5.279	0.000***	-0.151	-0.039
	Respect (authority/subversion)	-5.946	0.000***	-0.163	-0.051
	Competition (positive/negative)	-9.057	0.000***	-0.220	-0.107
	Fairness (fair/cheating)	-17.226	0.000***	-0.367	-0.254
	Purity (sanctity/degradation)	-7.057	0.000***	-0.184	-0.071
	Social-oriented (altruism/selfishness)	-10.947	0.000***	-0.253	-0.141

Table S2. Post-hoc comparisons for Target x Content interaction (Bonferroni-corrected for multiple comparisons). * p < 0.05, ** p < 0.01, *** p < 0.001.

Target	*Content*	*Content*	*t-value*	*p-value*	*95% Confidence interval for difference*	
					Lower bound	*Upper bound*
Ingroup	Prosociality (care/harm)	Community (loyalty/betrayal)	10.170	0.000***	0.220	0.414
		Respect (authority/subversion)	8.373	0.000***	0.164	0.359
		Competition (positive/negative)	6.448	0.000***	0.104	0.298
		Fairness (fair/cheating)	2.823	0.131	-0.009	0.186
		Purity (sanctity/degradation)	14.116	0.000***	0.342	0.537
		Social-oriented (altruism/selfishness)	3.144	0.047*	0.001	0.195
		General social affairs (positive/negative)	5.518	0.000***	0.074	0.269
	Community (loyalty/betrayal)	Prosociality (care/harm)	-10.170	0.000***	-0.414	-0.220
		Respect (authority/subversion)	-1.797	1.000	-0.153	0.042
		Competition (positive/negative)	-3.721	0.006**	-0.213	-0.019
		Fairness (fair/cheating)	-7.347	0.000***	-0.326	-0.131
		Purity (sanctity/degradation)	3.946	0.002**	0.025	0.220

		Social-oriented (altruism/selfishness)	-7.026	0.000***	-0.316	-0.122
		General social affairs (positive/negative)	-4.652	0.000***	-0.243	-0.048
	Respect (authority/subversion)	Prosociality (care/harm)	-8.373	0.000***	-0.359	-0.164
		Community (loyalty/betrayal)	1.797	1.000	-0.042	0.153
		Competition (positive/negative)	-1.925	1.000	-0.158	0.037
		Fairness (fair/cheating)	-5.550	0.000***	-0.271	-0.076
		Purity (sanctity/degradation)	5.711	0.000***	0.081	0.276
		Social-oriented (altruism/selfishness)	-5.229	0.000***	-0.261	-0.066
		General social affairs (positive/negative)	-2.887	0.111	-0.187	0.008
	Competition (positive/negative)	Prosociality (care/harm)	-6.448	0.000***	-0.298	-0.104
		Community (loyalty/betrayal)	3.721	0.006**	0.019	0.213
		Respect (authority/subversion)	1.925	1.000	-0.037	0.158
		Fairness (fair/cheating)	-3.625	0.008**	-0.210	-0.015
		Purity (sanctity/degradation)	7.668	0.000***	0.141	0.336
		Social-oriented (altruism/selfishness)	-3.304	0.027*	-0.200	-0.006

		General social affairs (positive/negative)	-0.930	1.000	-0.127	0.068
	Fairness (fair/cheating)	Prosociality (care/harm)	-2.823	0.131	-0.186	0.009
		Community (loyalty/betrayal)	7.347	0.000***	0.131	0.326
		Respect (authority/subversion)	5.550	0.000***	0.076	0.271
		Competition (positive/negative)	3.625	0.008**	0.015	0.210
		Purity (sanctity/degradation)	11.261	0.000***	0.254	0.449
		Social-oriented (altruism/selfishness)	0.321	1.000	-0.088	0.107
		General social affairs (positive/negative)	2.663	0.211	-0.014	0.181
	Purity (sanctity/degradation)	Prosociality (care/harm)	-14.116	0.000***	-0.537	-0.342
		Community (loyalty/betrayal)	-3.946	0.002**	-0.220	-0.025
		Respect (authority/subversion)	-5.711	0.000***	-0.276	-0.081
		Competition (positive/negative)	-7.668	0.000***	-0.336	-0.141
		Fairness (fair/cheating)	-11.261	0.000***	-0.449	-0.254
		Social-oriented (altruism/selfishness)	-10.972	0.000***	-0.439	-0.244
		General social affairs (positive/negative)	-8.598	0.000***	-0.365	-0.171

		Prosociality (care/harm)	-3.144	0.047*	-0.195	-0.001
		Community (loyalty/betrayal)	7.026	0.000***	0.122	0.316
		Respect (authority/subversion)	5.229	0.000***	0.066	0.261
	Social-oriented (altruism/selfishness)	Competition (positive/negative)	3.304	0.027*	0.006	0.200
		Fairness (fair/cheating)	-0.321	1.000	-0.107	0.088
		Purity (sanctity/degradation)	10.972	0.000***	0.244	0.439
		General social affairs (positive/negative)	2.374	0.514	-0.024	0.171
		Prosociality (care/harm)	-5.518	0.000***	-0.269	-0.074
		Community (loyalty/betrayal)	4.652	0.000***	0.048	0.243
		Respect (authority/subversion)	2.887	0.111	-0.008	0.187
	General social affairs (positive/negative)	Competition (positive/negative)	0.930	1.000	-0.068	0.127
		Fairness (fair/cheating)	-2.663	0.211	-0.181	0.014
		Purity (sanctity/degradation)	8.598	0.000***	0.171	0.365
		Social-oriented (altruism/selfishness)	-2.374	0.514	-0.171	0.024
Outgroup	Prosociality (care/harm)	Community (loyalty/betrayal)	17.517	0.000***	0.448	0.643

		Respect (authority/subversion)	14.212	0.000***	0.345	0.540
		Competition (positive/negative)	13.827	0.000***	0.334	0.529
		Fairness (fair/cheating)	9.240	0.000***	0.190	0.385
		Purity (sanctity/degradation)	11.100	0.000***	0.249	0.444
		Social-oriented (altruism/selfishness)	11.678	0.000***	0.267	0.462
		General social affairs (positive/negative)	19.410	0.000***	0.507	0.702
	Community (loyalty/betrayal)	Prosociality (care/harm)	-17.517	0.000***	-0.643	-0.448
		Respect (authority/subversion)	-3.304	0.027*	-0.200	-0.006
		Competition (positive/negative)	-3.657	0.007**	-0.212	-0.017
		Fairness (fair/cheating)	-8.277	0.000***	-0.356	-0.161
		Purity (sanctity/degradation)	-6.384	0.000***	-0.297	-0.102
		Social-oriented (altruism/selfishness)	-5.807	0.000***	-0.279	-0.084
		General social affairs (positive/negative)	1.893	1.000	-0.039	0.156
	Respect (authority/subversion)	Prosociality (care/harm)	-14.212	0.000***	-0.540	-0.345
		Community (loyalty/betrayal)	3.304	0.027*	0.006	0.200

		Competition (positive/negative)	-0.353	1.000	-0.109	0.086
		Fairness (fair/cheating)	-4.973	0.000***	-0.253	-0.058
		Purity (sanctity/degradation)	-3.080	0.056	-0.194	0.001
		Social-oriented (altruism/selfishness)	-2.502	0.333	-0.176	0.019
		General social affairs (positive/negative)	5.197	0.000***	0.064	0.259
	Competition (positive/negative)	Prosociality (care/harm)	-13.827	0.000***	-0.529	-0.334
		Community (loyalty/betrayal)	3.657	0.007**	0.017	0.212
		Respect (authority/subversion)	0.353	1.000	-0.086	0.109
		Fairness (fair/cheating)	-4.620	0.000***	-0.241	-0.046
		Purity (sanctity/degradation)	-2.727	0.180	-0.182	0.012
		Social-oriented (altruism/selfishness)	-2.149	0.887	-0.164	0.030
		General social affairs (positive/negative)	5.550	0.000***	0.076	0.271
	Fairness (fair/cheating)	Prosociality (care/harm)	-9.240	0.000***	-0.385	-0.190
		Community (loyalty/betrayal)	8.277	0.000***	0.161	0.356
		Respect (authority/subversion)	4.973	0.000***	0.058	0.253

		Competition (positive/negative)	4.620	0.000***	0.046	0.241
		Purity (sanctity/degradation)	1.893	1.000	-0.039	0.156
		Social-oriented (altruism/selfishness)	2.470	0.386	-0.021	0.174
		General social affairs (positive/negative)	10.170	0.000***	0.220	0.414
	Purity (sanctity/degradation)	Prosociality (care/harm)	-11.100	0.000***	-0.444	-0.249
		Community (loyalty/betrayal)	6.384	0.000***	0.102	0.297
		Respect (authority/subversion)	3.080	0.056	-0.001	0.194
		Competition (positive/negative)	2.727	0.180	-0.012	0.182
		Fairness (fair/cheating)	-1.893	1.000	-0.156	0.039
		Social-oriented (altruism/selfishness)	0.577	1.000	-0.079	0.115
		General social affairs (positive/negative)	8.277	0.000***	0.161	0.356
	Social-oriented (altruism/selfishness)	Prosociality (care/harm)	-11.678	0.000***	-0.462	-0.267
		Community (loyalty/betrayal)	5.807	0.000***	0.084	0.279
		Respect (authority/subversion)	2.502	0.333	-0.019	0.176
		Competition (positive/negative)	2.149	0.887	-0.030	0.164

		Fairness (fair/cheating)	-2.470	0.386	-0.174	0.021
		Purity (sanctity/degradation)	-0.577	1.000	-0.115	0.079
		General social affairs (positive/negative)	7.700	0.000***	0.143	0.338
	General social affairs (positive/negative)	Prosociality (care/harm)	-19.410	0.000***	-0.702	-0.507
		Community (loyalty/betrayal)	-1.893	1.000	-0.156	0.039
		Respect (authority/subversion)	-5.197	0.000***	-0.259	-0.064
		Competition (positive/negative)	-5.550	0.000***	-0.271	-0.076
		Fairness (fair/cheating)	-10.170	0.000***	-0.414	-0.220
		Purity (sanctity/degradation)	-8.277	0.000***	-0.356	-0.161
		Social-oriented (altruism/selfishness)	-7.700	0.000***	-0.338	-0.143
Celebrities	Prosociality (care/harm)	Community (loyalty/betrayal)	8.855	0.000***	0.179	0.374
		Respect (authority/subversion)	12.801	0.000***	0.301	0.496
		Competition (positive/negative)	9.657	0.000***	0.203	0.398
		Fairness (fair/cheating)	3.721	0.006**	0.019	0.213
		Purity (sanctity/degradation)	8.181	0.000***	0.157	0.352

		Social-oriented (altruism/selfishness)	11.838	0.000***	0.272	0.467
		General social affairs (positive/negative)	20.757	0.000***	0.550	0.744
	Community (loyalty/betrayal)	Prosociality (care/harm)	-8.855	0.000***	-0.374	-0.179
		Respect (authority/subversion)	3.946	0.002**	0.025	0.220
		Competition (positive/negative)	0.802	1.000	-0.073	0.122
		Fairness (fair/cheating)	-5.133	0.000***	-0.258	-0.063
		Purity (sanctity/degradation)	-0.674	1.000	-0.119	0.076
		Social-oriented (altruism/selfishness)	2.984	0.079	-0.004	0.191
		General social affairs (positive/negative)	11.902	0.000***	0.273	0.468
	Respect (authority/subversion)	Prosociality (care/harm)	-12.801	0.000***	-0.496	-0.301
		Community (loyalty/betrayal)	-3.946	0.002**	-0.220	-0.025
		Competition (positive/negative)	-3.144	0.047*	-0.195	-0.001
		Fairness (fair/cheating)	-9.079	0.000***	-0.380	-0.185
		Purity (sanctity/degradation)	-4.620	0.000***	-0.241	-0.046
		Social-oriented (altruism/selfishness)	-0.930	1.000	-0.127	0.068

		General social affairs (positive/negative)	7.956	0.000***	0.151	0.346
	Competition (positive/negative)	Prosociality (care/harm)	-9.657	0.000***	-0.398	-0.203
		Community (loyalty/betrayal)	-0.802	1.000	-0.122	0.073
		Respect (authority/subversion)	3.144	0.047*	0.001	0.195
		Fairness (fair/cheating)	-5.935	0.000***	-0.282	-0.087
		Purity (sanctity/degradation)	-1.476	1.000	-0.143	0.052
		Social-oriented (altruism/selfishness)	2.214	0.777	-0.029	0.166
		General social affairs (positive/negative)	11.100	0.000***	0.249	0.444
	Fairness (fair/cheating)	Prosociality (care/harm)	-3.721	0.006**	-0.213	-0.019
		Community (loyalty/betrayal)	5.133	0.000***	0.063	0.258
		Respect (authority/subversion)	9.079	0.000***	0.185	0.380
		Competition (positive/negative)	5.935	0.000***	0.087	0.282
		Purity (sanctity/degradation)	4.459	0.000***	0.041	0.236
		Social-oriented (altruism/selfishness)	8.117	0.000***	0.156	0.351
		General social affairs (positive/negative)	17.035	0.000***	0.434	0.628

	Purity (sanctity/degradation)	Prosociality (care/harm)	-8.181	0.000***	-0.352	-0.157
		Community (loyalty/betrayal)	0.674	1.000	-0.076	0.119
		Respect (authority/subversion)	4.620	0.000***	0.046	0.241
		Competition (positive/negative)	1.476	1.000	-0.052	0.143
		Fairness (fair/cheating)	-4.459	0.000***	-0.236	-0.041
		Social-oriented (altruism/selfishness)	3.657	0.007**	0.017	0.212
		General social affairs (positive/negative)	12.576	0.000***	0.295	0.490
	Social-oriented (altruism/selfishness)	Prosociality (care/harm)	-11.838	0.000***	-0.467	-0.272
		Community (loyalty/betrayal)	-2.984	0.079	-0.191	0.004
		Respect (authority/subversion)	0.930	1.000	-0.068	0.127
		Competition (positive/negative)	-2.214	0.777	-0.166	0.029
		Fairness (fair/cheating)	-8.117	0.000***	-0.351	-0.156
		Purity (sanctity/degradation)	-3.657	0.007**	-0.212	-0.017
		General social affairs (positive/negative)	8.919	0.000***	0.180	0.375
	General social affairs (positive/negative)	Prosociality (care/harm)	-20.757	0.000***	-0.744	-0.550

		Community (loyalty/betrayal)	-11.902	0.000***	-0.468	-0.273
		Respect (authority/subversion)	-7.956	0.000***	-0.346	-0.151
		Competition (positive/negative)	-11.100	0.000***	-0.444	-0.249
		Fairness (fair/cheating)	-17.035	0.000***	-0.628	-0.434
		Purity (sanctity/degradation)	-12.576	0.000***	-0.490	-0.295
		Social-oriented (altruism/selfishness)	-8.919	0.000***	-0.375	-0.180

Table S3. Post-hoc comparisons for Target x Valence x Content interaction (Bonferroni-corrected for multiple comparisons). * p < 0.05, ** p < 0.01, *** p < 0.001.

Target-by-valence	*Content*	*Content*	*t-value*	*p-value*	*95% Confidence interval for difference*	
					Lower bound	*Upper bound*
Celebrity - Negative	Prosociality (harm)	Community (betrayal)	6.896	0.000***	0.166	0.442
		Respect (subversion)	6.238	0.000***	0.137	0.412
		Competition (negative)	3.788	0.004**	0.029	0.304
		Fairness (cheating)	1.044	1.000	-0.092	0.184
		Purity (degradation)	2.518	0.329	-0.027	0.249
		Social-oriented (selfishness)	8.235	0.000***	0.225	0.501
		General social affairs (negative)	13.725	0.000***	0.467	0.742
	Community (betrayal)	Prosociality (harm)	-6.896	0.000***	-0.442	-0.166
		Respect (subversion)	-0.658	1.000	-0.167	0.108
		Competition (negative)	-3.108	0.052	-0.275	0.001
		Fairness (cheating)	-5.853	0.000***	-0.396	-0.120
		Purity (degradation)	-4.378	0.000***	-0.331	-0.055
		Social-oriented (selfishness)	1.338	1.000	-0.079	0.197
		General social affairs (negative)	6.828	0.000***	0.163	0.438
	Respect (subversion)	Prosociality (harm)	-6.238	0.000***	-0.412	-0.137
		Community (betrayal)	0.658	1.000	-0.108	0.167

		Competition (negative)	-2.450	0.405	-0.246	0.030
		Fairness (cheating)	-5.195	0.000***	-0.367	-0.091
		Purity (degradation)	-3.698	0.006**	-0.301	-0.026
		Social-oriented (selfishness)	1.996	1.000	-0.050	0.226
		General social affairs (negative)	7.486	0.000***	0.192	0.468
	Competition (negative)	Prosociality (harm)	-3.788	0.004**	-0.304	-0.029
		Community (betrayal)	3.108	0.052	-0.001	0.275
		Respect (subversion)	2.450	0.405	-0.030	0.246
		Fairness (cheating)	-2.745	0.171	-0.259	0.017
		Purity (degradation)	-1.270	1.000	-0.193	0.082
		Social-oriented (selfishness)	4.446	0.000***	0.058	0.334
		General social affairs (negative)	9.936	0.000***	0.300	0.576
	Fairness (cheating)	Prosociality (harm)	-1.044	1.000	-0.184	0.092
		Community (betrayal)	5.853	0.000***	0.120	0.396
		Respect (subversion)	5.195	0.000***	0.091	0.367
		Competition (negative)	2.745	0.171	-0.017	0.259
		Purity (degradation)	1.475	1.000	-0.072	0.203
		Social-oriented (selfishness)	7.191	0.000***	0.179	0.455
		General social affairs (negative)	12.681	0.000***	0.421	0.697

	Purity (degradation)	Prosociality (harm)	-2.518	0.329	-0.249	0.027
		Community (betrayal)	4.378	0.000***	0.055	0.331
		Respect (subversion)	3.698	0.006**	0.026	0.301
		Competition (negative)	1.270	1.000	-0.082	0.193
		Fairness (cheating)	-1.475	1.000	-0.203	0.072
		Social-oriented (selfishness)	5.717	0.000***	0.114	0.389
		General social affairs (negative)	11.184	0.000***	0.356	0.631
	Social-oriented (selfishness)	Prosociality (harm)	-8.235	0.000***	-0.501	-0.225
		Community (betrayal)	-1.338	1.000	-0.197	0.079
		Respect (subversion)	-1.996	1.000	-0.226	0.050
		Competition (negative)	-4.446	0.000***	-0.334	-0.058
		Fairness (cheating)	-7.191	0.000***	-0.455	-0.179
		Purity (degradation)	-5.717	0.000***	-0.389	-0.114
		General social affairs (negative)	5.490	0.000***	0.104	0.380
	General social affairs (negative)	Prosociality (harm)	-13.725	0.000***	-0.742	-0.467
		Community (betrayal)	-6.828	0.000***	-0.438	-0.163
		Respect (subversion)	-7.486	0.000***	-0.468	-0.192
		Competition (negative)	-9.936	0.000***	-0.576	-0.300
		Fairness (cheating)	-12.681	0.000***	-0.697	-0.421
		Purity (degradation)	-11.184	0.000***	-0.631	-0.356

		Social-oriented (selfishness)	-5.490	0.000***	-0.380	-0.104
Outgroup - Negative	Prosociality (harm)	Community (betrayal)	11.343	0.000***	0.362	0.638
		Respect (subversion)	7.554	0.000***	0.196	0.471
		Competition (negative)	9.414	0.000***	0.277	0.553
		Fairness (cheating)	7.032	0.000***	0.173	0.448
		Purity (degradation)	3.494	0.014*	0.016	0.291
		Social-oriented (selfishness)	11.048	0.000***	0.349	0.625
		General social affairs (negative)	13.112	0.000***	0.441	0.716
	Community (betrayal)	Prosociality (harm)	-11.343	0.000***	-0.638	-0.362
		Respect (subversion)	-3.788	0.004**	-0.304	-0.029
		Competition (negative)	-1.928	1.000	-0.223	0.053
		Fairness (cheating)	-4.310	0.000***	-0.327	-0.052
		Purity (degradation)	-7.849	0.000***	-0.484	-0.209
		Social-oriented (selfishness)	-0.295	1.000	-0.151	0.125
		General social affairs (negative)	1.769	1.000	-0.059	0.216
	Respect (subversion)	Prosociality (harm)	-7.554	0.000***	-0.471	-0.196
		Community (betrayal)	3.788	0.004**	0.029	0.304
		Competition (negative)	1.860	1.000	-0.056	0.219
		Fairness (cheating)	-0.522	1.000	-0.161	0.115

		Purity (degradation)	-4.083	0.001**	-0.318	-0.042
		Social-oriented (selfishness)	3.494	0.014*	0.016	0.291
		General social affairs (negative)	5.558	0.000***	0.107	0.383
	Competition (negative)	Prosociality (harm)	-9.414	0.000***	-0.553	-0.277
		Community (betrayal)	1.928	1.000	-0.053	0.223
		Respect (subversion)	-1.860	1.000	-0.219	0.056
		Fairness (cheating)	-2.382	0.496	-0.242	0.033
		Purity (degradation)	-5.921	0.000***	-0.399	-0.124
		Social-oriented (selfishness)	1.633	1.000	-0.066	0.210
		General social affairs (negative)	3.698	0.006**	0.026	0.301
	Fairness (cheating)	Prosociality (harm)	-7.032	0.000***	-0.448	-0.173
		Community (betrayal)	4.310	0.000***	0.052	0.327
		Respect (subversion)	0.522	1.000	-0.115	0.161
		Competition (negative)	2.382	0.496	-0.033	0.242
		Purity (degradation)	-3.562	0.011*	-0.295	-0.019
		Social-oriented (selfishness)	3.993	0.002**	0.039	0.314
		General social affairs (negative)	6.080	0.000***	0.130	0.406
	Purity (degradation)	Prosociality (harm)	-3.494	0.014*	-0.291	-0.016
		Community (betrayal)	7.849	0.000***	0.209	0.484

		Respect (subversion)	4.083	0.001**	0.042	0.318
		Competition (negative)	5.921	0.000***	0.124	0.399
		Fairness (cheating)	3.562	0.011*	0.019	0.295
		Social-oriented (selfishness)	7.554	0.000***	0.196	0.471
		General social affairs (negative)	9.641	0.000***	0.287	0.563
	Social-oriented (selfishness)	Prosociality (harm)	-11.048	0.000***	-0.625	-0.349
		Community (betrayal)	0.295	1.000	-0.125	0.151
		Respect (subversion)	-3.494	0.014*	-0.291	-0.016
		Competition (negative)	-1.633	1.000	-0.210	0.066
		Fairness (cheating)	-3.993	0.002**	-0.314	-0.039
		Purity (degradation)	-7.554	0.000***	-0.471	-0.196
		General social affairs (negative)	2.087	1.000	-0.046	0.229
	General social affairs (negative)	Prosociality (harm)	-13.112	0.000***	-0.716	-0.441
		Community (betrayal)	-1.769	1.000	-0.216	0.059
		Respect (subversion)	-5.558	0.000***	-0.383	-0.107
		Competition (negative)	-3.698	0.006**	-0.301	-0.026
		Fairness (cheating)	-6.080	0.000***	-0.406	-0.130
		Purity (degradation)	-9.641	0.000***	-0.563	-0.287
		Social-oriented (selfishness)	-2.087	1.000	-0.229	0.046
	Prosociality (harm)	Community (betrayal)	1.475	1.000	-0.072	0.203

		Respect (subversion)	2.223	0.733	-0.040	0.236
		Competition (negative)	1.701	1.000	-0.063	0.213
		Fairness (cheating)	-0.454	1.000	-0.157	0.118
		Purity (degradation)	4.151	0.001**	0.045	0.321
		Social-oriented (selfishness)	-0.159	1.000	-0.144	0.131
		General social affairs (negative)	-1.112	1.000	-0.187	0.089
	Community (betrayal)	Prosociality (harm)	-1.475	1.000	-0.203	0.072
		Respect (subversion)	0.749	1.000	-0.105	0.170
		Competition (negative)	0.227	1.000	-0.128	0.148
Ingroup - Negative		Fairness (cheating)	-1.928	1.000	-0.223	0.053
		Purity (degradation)	2.677	0.214	-0.020	0.255
		Social-oriented (selfishness)	-1.633	1.000	-0.210	0.066
		General social affairs (negative)	-2.586	0.266	-0.252	0.023
	Respect (subversion)	Prosociality (harm)	-2.223	0.733	-0.236	0.040
		Community (betrayal)	-0.749	1.000	-0.170	0.105
		Competition (negative)	-0.522	1.000	-0.161	0.115
		Fairness (cheating)	-2.677	0.214	-0.255	0.020
		Purity (degradation)	1.928	1.000	-0.053	0.223
		Social-oriented (selfishness)	-2.382	0.496	-0.242	0.033

		General social affairs (negative)	-3.335	0.024*	-0.285	-0.009
	Competition (negative)	Prosociality (harm)	-1.701	1.000	-0.213	0.063
		Community (betrayal)	-0.227	1.000	-0.148	0.128
		Respect (subversion)	0.522	1.000	-0.115	0.161
		Fairness (cheating)	-2.155	0.885	-0.233	0.043
		Purity (degradation)	2.450	0.405	-0.030	0.246
		Social-oriented (selfishness)	-1.860	1.000	-0.219	0.056
		General social affairs (negative)	-2.813	0.136	-0.262	0.014
	Fairness (cheating)	Prosociality (harm)	0.454	1.000	-0.118	0.157
		Community (betrayal)	1.928	1.000	-0.053	0.223
		Respect (subversion)	2.677	0.214	-0.020	0.255
		Competition (negative)	2.155	0.885	-0.043	0.233
		Purity (degradation)	4.605	0.000***	0.065	0.340
		Social-oriented (selfishness)	0.295	1.000	-0.125	0.151
		General social affairs (negative)	-0.658	1.000	-0.167	0.108
	Purity (degradation)	Prosociality (harm)	-4.151	0.001**	-0.321	-0.045
		Community (betrayal)	-2.677	0.214	-0.255	0.020
		Respect (subversion)	-1.928	1.000	-0.223	0.053
		Competition (negative)	-2.450	0.405	-0.246	0.030

		Fairness (cheating)	-4.605	0.000***	-0.340	-0.065
		Social-oriented (selfishness)	-4.310	0.000***	-0.327	-0.052
		General social affairs (negative)	-5.263	0.000***	-0.370	-0.094
	Social-oriented (selfishness)	Prosociality (harm)	0.159	1.000	-0.131	0.144
		Community (betrayal)	1.633	1.000	-0.066	0.210
		Respect (subversion)	2.382	0.496	-0.033	0.242
		Competition (negative)	1.860	1.000	-0.056	0.219
		Fairness (cheating)	-0.295	1.000	-0.151	0.125
		Purity (degradation)	4.310	0.000***	0.052	0.327
		General social affairs (negative)	-0.953	1.000	-0.180	0.095
	General social affairs (negative)	Prosociality (harm)	1.112	1.000	-0.089	0.187
		Community (betrayal)	2.586	0.266	-0.023	0.252
		Respect (subversion)	3.335	0.024*	0.009	0.285
		Competition (negative)	2.813	0.136	-0.014	0.262
		Fairness (cheating)	0.658	1.000	-0.108	0.167
		Purity (degradation)	5.263	0.000***	0.094	0.370
		Social-oriented (selfishness)	0.953	1.000	-0.095	0.180

3 More positive gossiping or less negative gossiping for ingroup targets

Here we examine the three-way interaction (Content x Valence x Target) results in more detail, focusing on potential evidence for whether skewing positive for ingroup targets results from more positive gossiping or less negative gossiping (i.e., elevated positivity or avoided negativity). There was more positive gossiping for *respect* (*authority)*, *competition* (*positive*) *competition*, and social-oriented (*altruism*) compared to both celebrities and outgroup (Fig S4I, K, M & O), suggesting heightened positivity for these content domains. (As well as more overall gossiping of *general social affairs* for ingroup members: Fig S4O & P.)

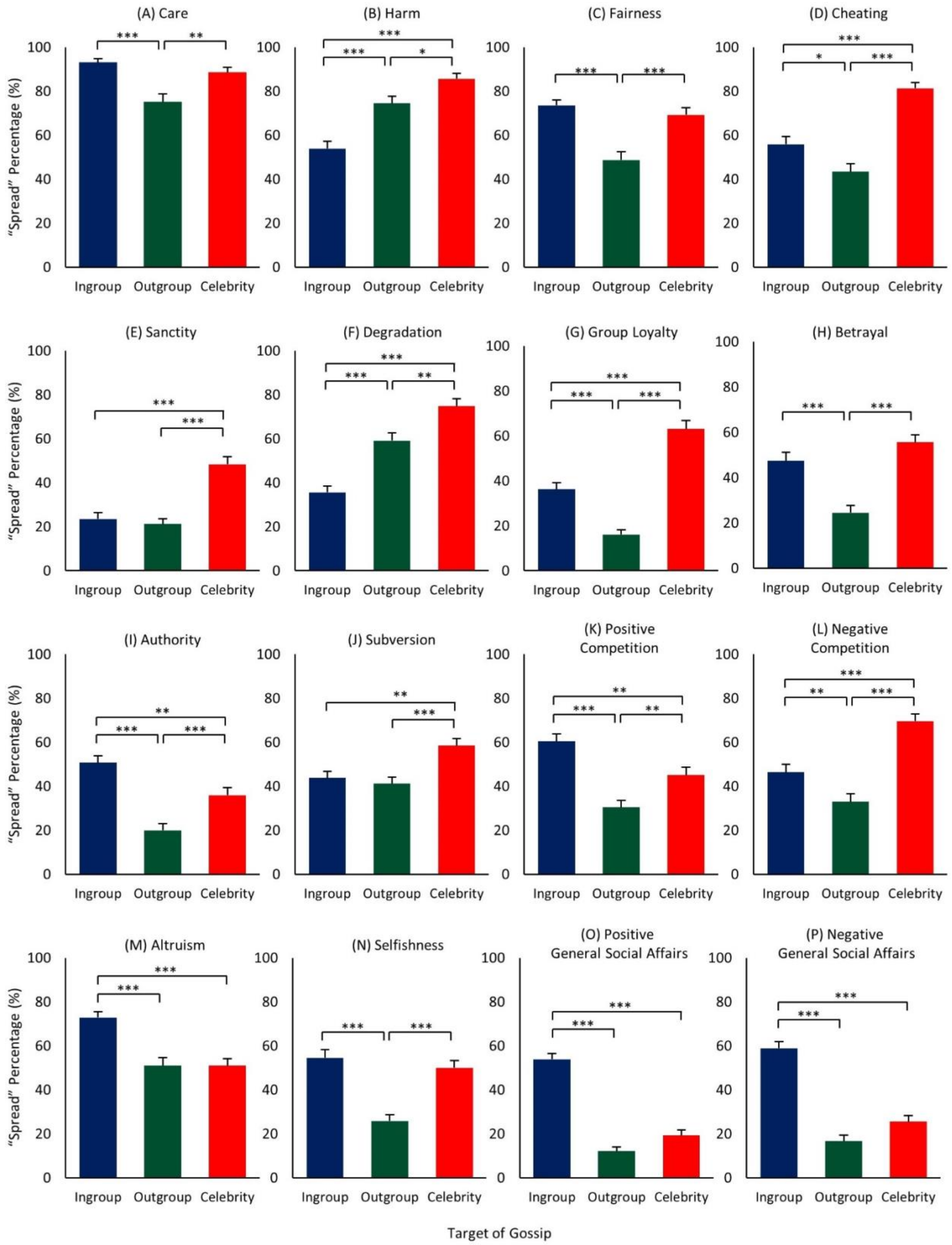

Fig S4. Mean gossip spread rates of 3-way interaction (Content x Valence x Target). Each graph shows the mean spread rates of ingroup, outgroup, and celebrity gossip for 16 different pairs of valence and content domains labelled at top of each graph. * $p < 0.05$, ** $p < 0.01$, *** $p < 0.001$.

Whereas for *care/harm* and *fair/cheating*, negative gossiping about ingroup targets was significantly less than that for both celebrities and outgroup targets for *harm* scenarios (Fig S4B), and significantly less than celebrities and closer to outgroup spreading rates for *cheating* (Fig S4D). In fact, an examination of all such differences across content domains suggests potential dampening of negative gossiping for ingroup targets with *harm*, *degradation* (as well as dampening of *sanctity*), *subversion*, and possibly *cheating* and *negative competition* (Figs S4B, D, E, F, J & L). This lowering of negative ingroup gossiping suggests that these content topics (i.e., with negative valence) were being avoided.

4 Negativity-avoidance effect for ingroup targets

Here we address the questions of why certain topics were avoided, and why there was a general negativity-avoidance effect for ingroup targets (Fig 9). Less negativity with ingroup targets might generally be suspected, but this effect is potentially problematic with respect to social checks and balances. Are ingroup members generally permitted to commit harm and cheat the system, for example? We consider four possibilities of why certain topics were avoided, and why there was a general negativity-avoidance effect for ingroup targets.

First, perhaps people would indeed prefer to 'punish' the target individual (to correct their behavior) or protect others (by updating beliefs of possible receivers), however, the possibility of detrimental repercussions may be too high, including harm to the target person and/or the gossiper him/herself (e.g., with respect to reputation and relationships with others) [see 6, 47]. We suspect that this factor may play a significant role in the findings.

Second, people may actually not wish to punish those in the ingroup via gossiping and social reputation. This could be due to having other means to police the ingroup: e.g., direct interaction with the target person, using positive versus negative means of correcting behavior. This possibility also

seems quite likely to play an important role. Although others have found that direct punishment may be less effective than social reputation manipulation [20], the relative merits of direct vs. indirect punishment and communication likely depend on specific circumstances, and thus would be ripe for computational modeling to specify the circumstances.

Third, people may be generally less sensitive about certain matters such as hierarchy and rankings among friends — i.e., ingroup members may be considered generally of equal status. Indeed, our participants may consider themselves relatively lower in status (especially being students of 20-32, mean 23.8, years of age). This relatively lower and similar ranking may allow some license to the target individual for borderline behaviors, such as perhaps more selfishness and cheating if these are construed as not too egregious. We wouldn't expect this possibility to entirely explain the results, however, given that such problem behaviors even from one's ingroup would generally be expected to concern people.

And fourth, there is likely greater empathy for ingroup members (due to, e.g., more frequent interactions, closer sense of similarities, etc.), and thus a stronger awareness of the effects the gossiping may have on the target (e.g., knowing more clearly that you are making fun of, embarrassing, and/or potentially harming the target's reputation). We again, however, would not expect this possibility to entirely explain the results.

5 Negative gossiping about celebrities and relationship to status

We next provide a closer examination of the possible reasons underlying the heightened negative gossiping about celebrities. Negative gossiping may derive from a desire to (i) punish the target (to correct their behavior), (ii) protect receivers (by notifying them of aberrant behavior), (iii) manage the social hierarchy via raising one's own and perhaps the receiver's relative status by lowering that of the celebrity (at least within the gossiper's social reach), or (iv) perhaps due simply to intrigue, if such negative scenarios prove especially interesting (i.e., being beyond typical expectations).

Heightened negative gossiping suggests either an even stronger desire for one or more of possibilities (i-iv) or simply the fact that there may be lower risk of negative repercussions to the gossiper (when gossiping about celebrities or outgroup strangers). The lower risk of negative ramifications does seem likely to be a significant factor driving more negative gossiping of celebrities; however, because the lower risk would seem comparable or even less for outgroup targets, and yet gossiping in general is lower about them, possible backlash does not appear to be the only driving factor (Table 1).

For possibility (iv), intrigue, first, in participant ratings of interest level, we did not find a significant difference of negative valence being gossiped more than positive; and in fact, we found the opposite, though negative were more emotionally evocative (see *Participant Ratings* in main text). Nonetheless, intrigue with respect to garnering interest by being out-of-the norm should also occur for ingroup targets too, so the higher degree with celebrities does not seem to be driven largely by general interest, though further research to tease apart and clarify these effects is warranted.

In fact, possibilities (i)-(iii) (punish, protect, manage hierarchy) all appear to relate to status. Regarding possibility (i), punishment of target, it would suggest that the greater desire to punish celebrity targets would stem from their higher profile and status, either due to holding those above you to higher standards to justify the inequality or lowering their higher status when possible. With respect to specifically attempting to punish them with gossip, although there may be some sense of this, especially with the possible reach of social media, it nonetheless seems less likely to be much of a significant factor, with celebrities representing more of a one-way interaction. However, although punishment via negative gossiping may not actually affect the target celebrity (due to lack of complete bi-directional interaction), the action of spreading gossip itself may relieve a certain desire of a person (i.e., a gossiper) seeking justice. That is, taking an action of punishment via negative gossiping, regardless of the actual punishment and its consequences, may provide some level of

satisfaction (and thus motivate people to instigate gossip). Furthermore, by spreading negative gossip about a celebrity, a gossiper can convey a message to the general population that the targeted behavior is not socially acceptable and thus needed to be held accountable.

For possibility (ii), protection, given that celebrities likely influence those who are interested in them (e.g., as role models), it in turn seems likely that one would wish to notify others when evidence suggests that people's views of the celebrity should be updated based on particularly negative actions undertaken by them (correcting false beliefs). Protection, then, seems likely to be an important component. Related to possibilities (i) and (ii), possibility (iii), social hierarchy management within one's social range, also appears to be a viable possibility if the gossiper hopes to raise his/her own and perhaps the receivers' status as well by lowering that of the celebrity, at least among those within the gossiper's purview, which of course would be among those expected to be most relevant to the gossiper.

6 Overall effects of status

Indeed, a closer examination of the content domains further suggests that status played an important role in the potential gossiper's decision of whether to spread the news about them. For higher-status celebrities, *harm*, *cheating*, *loyalty*, *subversion*, and *negative competition* were particularly important (along with *degradation* as discussed separately) (Figs 9C & E, Fig 12). These content topics suggest that a show of loyalty and humility at higher status warrants interest (perhaps 'warms peoples' hearts'), whereas trying to beat others in harmful or underhanded ways, or otherwise cheating the system is especially bothersome for celebrity targets, presumably because they represent higher status people (and thus apparently less so for those at lower status levels — i.e., ingroup and outgroup).

Moreover, the greater interest in *fairness* (over *cheating*) and *altruism* (over *selfishness*) for both ingroup and outgroup targets suggests that such fair and selfless acts are especially impressive when conducted by those of lesser status and means (Figs 10 & 11). Put differently, people may

believe that celebrities (as representatives of our society) need to be altruistic and selfless; therefore, altruistic/selfless information about celebrities is considered to be less worthy of spreading. Whereas, altruistic/selfless behavior of ingroup and outgroup targets (who have similar or possibly lower status compared to the gossiper) impresses people (i.e., possible gossipers) more than the same type of information about celebrities, and people consider such information (i.e., altruistic behaviors of ingroup and outgroup members) worthwhile to spread. At the same time, selfish behavior of ingroup and outgroup people are relatively less spread. This finding suggests that such acts may be more tolerated in those with less means and status.

Finally, the higher gossiping rate for *authority* and *positive competition* with ingroup targets (compared to the other target groups; Figs 9I & K) suggests that these topics are more relevant and interesting among relatively lower (compared to celebrities) and more equal status ingroup members, with *positive competition* suggesting scenarios of ambition or achievement resonate more with those striving to improve their status and perhaps especially with friends whose achievements may benefit the entire ingroup.
